# How Does Domestic Cooking Affect the Biochemical Properties of Wild Edible Greens of the Asteraceae Family?

**DOI:** 10.3390/foods13172677

**Published:** 2024-08-25

**Authors:** Vasiliki Liava, Ângela Fernandes, Filipa Reis, Tiane Finimundy, Filipa Mandim, José Pinela, Dejan Stojković, Isabel C. F. R. Ferreira, Lillian Barros, Spyridon A. Petropoulos

**Affiliations:** 1 Laboratory of Vegetable Production, University of Thessaly, Fytokou Street, 38446 Volos, Greece; vasiliki.liava@gmail.com; 2 Centro de Investigação de Montanha (CIMO), Instituto Politécnico de Bragança, Campus de Santa Apolónia, 5300-253 Bragança, Portugal; freis@ipb.pt (F.R.); tiane@ipb.pt (T.F.); filipamandim@ipb.pt (F.M.); jpinela@ipb.pt (J.P.); iferreira@ipb.pt (I.C.F.R.F.); lillian@ipb.pt (L.B.); 3 Laboratório Associado para a Sustentabilidade e Tecnologia em Regiões de Montanha (SusTEC), Instituto Politécnico de Bragança, 5300-253 Bragança, Portugal; 4 Department of Plant Physiology, Institute for Biological Research “Siniša Stanković”-National Institute of Republic of Serbia, University of Belgrade, Bulevar Despota Stefana 142, 11000 Belgrade, Serbia; dejanbio@ibiss.bg.ac.rs

**Keywords:** wild edible species, bioactive properties, decoctions, nutritional profile, organic acids, antimicrobial properties

## Abstract

Wild edible greens are a key ingredient of the so-called Mediterranean diet and they are commonly used in various local dishes in their raw or processed form. Domestic processing of edible greens may affect their nutritional value and chemical profile. In this work, six wild species (e.g., *Cichorium spinosum* L. (S1); *Centaurea raphanina* subsp. *mixta* (DC.) Runemark (S2); *Picris echioides* (L.) Holub (S3); *Urospermum picroides* (L.) Scop. ex. F.W. Schmidt (S4); *Sonchus oleraceus* L. (S5); and *S. asper* L. (S6)) were assessed for the effect of domestic processing (boiling) on chemical composition and bioactivities. Concerning the chemical composition, glucose, oxalic acid, α-tocopherol, and α-linolenic acid were the most abundant compounds, especially in *P. echiodes* leaves. After decoction, mainly sugars, tocopherols, and oxalic acid were decreased. The species and processing affected the phenolic compounds content and antioxidant, cytotoxicity, and anti-inflammatory activities. Specific compounds were not previously detected in the studied species, while hydroethanolic extracts contained a higher total phenolic compound content. Hydroethanolic and aqueous extracts were effective towards a range of bacterial and fungi strains. Therefore, the consumption of leaves has health-promoting properties owing to the bioactive compounds and can be integrated into healthy diets. However, domestic cooking may affect the chemical profile and bioactivities of the edible leaves, especially in the case of free sugars and phenolic compound content where a significant reduction was recorded in leaves after decoction. On the other hand, domestic processing could be beneficial since it reduces the oxalic acid content in edible leaves, which is considered an antinutritional factor.

## 1. Introduction

Wild edible plants are grown without human intervention using the available natural resources [1]. During recent years, scientific attention has shifted to these plants and several studies have been conducted assessing their therapeutic and nutritional properties [2] while their consumption has been gradually increasing as people search for healthy and functional food sources [3]. Recent studies in Greece indicated that *Silybum marianum* (L.) Gaertn (milk thistle) and *Portulaca oleracea* L. (common purslane), which are usually found as weeds, can be introduced as alternative/complementary crops in small-scale farming systems [4,5,6]. Wild plants are attributed with high resistance to abiotic stress and their commercial exploitation could facilitate the Sustainable Development Goals (SDGs) suggested by the UN and the current EU policy regarding environmentally friendly practices in crop production [7,8,9,10]. Southern Europe and the broader Mediterranean Basin are abundant with wild edible species which have remarkable nutritional and medicinal value [11] and constitute a rich dietary source of phytochemicals (secondary metabolites) [12,13,14]. Although plant secondary metabolites are commonly not vital to sustain human life, recent experimental works have shown that they have significant beneficial health effects [15,16]. Wild plants usually contain numerous plant secondary metabolites such as vitamin E and C, phenolic compounds, pigments such as carotenoids and anthocyanins, and terpenoids, which contribute to their antioxidant capacity [1]. Therefore, there is high potential to valorize these unexploited species, which are considered as noxious weeds in many crops, as “novel functional foods” in diversified diets [1,11].

The Asteraceae family consists of many wild edible species with high nutritional and nutraceutical properties which are an essential part of the Mediterranean diet [17]. “Stamnagathi” or spiny chicory (*Cichorium spinosum* L.), a wild chicory species with important health-promoting properties, is a very adaptive wild plant that grows in various regions in the Mediterranean Basin [18]. Its commercial cultivation has been promoted over the last few years as an alternative vegetable crop [19]. *Centaurea raphanina* subsp. *mixta* is another edible herb endemic to Greece, which is commonly known as “alibarbaron” or “agginaráki” [20], that can grow under striving conditions, including high altitudes, rocky areas, and low temperatures [21]. Bristly oxtongue (*Helminthotheca echioides* L.) is a common weed in several cereal crops, and its leaves are commonly consumed in the Mediterranean diet [22,23,24]. *Urospermum picroides* L. (prickly golden fleece) grows under harsh conditions and is commonly consumed in the Mediterranean region in various local dishes [25,26,27]. The extracts of this plant have also revealed important biological effects, including antioxidant, antiproliferative, anti-inflammatory, and antidiabetic activities [28]. Finally, the *Sonchus* genus comprises about 60 species commonly found in many regions of the world, including several common weeds and wild edible plants such as *S. oleraceus* L. (common sow thistle) and *S. asper* L. (prickly sow thistle) [29,30,31]. Both species are rich in phenolic compounds, carotenoids, and vitamins and are highly appreciated in local cuisines [29,30].

Wild edible plants are commonly eaten fresh (raw), boiled, cooked, or following other domestic processing [13,32], while fresh and dried herbs can also be prepared as beverages and herbal teas (decoctions) with several beneficial effects on health due to their phytochemical content [33]. In herbal remedies, whole plants, underground parts (roots, bulbs, tubers, rhizomes), fruit, seeds, stems, and flowers can be used, although leaves are the most commonly used plant part [34]. In contrast, conventional extraction methods, e.g., digestion, maceration, infusion, percolation, and decoction, are widely used by the scientific community to reveal the chemical profile and the bioactivities of these species [35]. Decoction is among the oldest and most popular methods for herbal medicine preparation, since it is an easy technique allowing the extraction of plant compounds in boiled water [34]. Moreover, decoction preparations may ensure out-of-season product availability, as well as additional high-added-value end-products for industry sectors [36]. Although most scientific reports suggest significant health effects for the extracts or the edible tissues in fresh or dried form, there is scarce information regarding the impact of domestic processing on the bioactive properties and chemical profile of wild edible greens [22,37].

Therefore, the goals of this work were to determine the proximate composition and chemical and bioactive properties of six wild edible greens before and after the decoction (boiling) process, as well as those of the decoction water, aiming to assess the impact of a common domestic processing method on the quality of the edible product. Moreover, our work aimed to reveal the potential of using decoction water as a source of valuable bioactive phytochemicals with further uses in industry sectors.

## 2. Materials and Methods

### 2.1. Standards and Reagents

HPLC-grade acetonitrile (99.9%) was purchased from Fisher Scientific (Lisbon, Portugal). The fatty acid methyl ester (FAME) reference standard mixture 37 (standard 47885-U) [38], sugars, organic acids, E211, E224, and Trolox (6-hydroxy-2,5,7,8-tetramethylchroman-2-carboxylic acid) were purchased from Sigma (St. Louis, MO, USA). Tocopherol standards and Tocol were purchased from Matreya (Pleasant Gap, PA, USA). Formic and acetic acids were purchased from Prolabo (VWR International, Briare, France). Ethyl acetate (99.8%) was purchased from Fisher Scientific (Lisbon, Portugal). Phenolic compound standards were purchased from Extrasynthese (Genay, France). Fetal bovine serum (FBS), L-glutamine, Hank’s balanced salt solution (HBSS), and trypsin–EDTA (ethyl-enediaminetetraacetic acid) were purchased from Hyclone (Logan, UT, USA). Acetic acid, ellipticine, sulforhodamine B (SRB), trypan blue, tri-chloroacetic acid (TCA), and tris (tris(hydroxymethyl)amino-methane) were purchased from Sigma Chemical Co. (St. Louis, MO, USA). The cell lines CaCo2 (Catalog No. 860102022) and RAW 264.7 (Catalog No. 91062702) were commercially acquired from the European Collection of Authenticated Cell Cultures—ECACC; in turn, NCI-H460 (ACC 737) and MCF-7 (ACC 115) were acquired from the Leibniz Institute DSMZ—German Collection of Microorganisms and Cell Cultures GmbH. Mueller–Hinton agar (MH) and malt agar (MA) were obtained from the Institute of Immunology and Virology, Torlak (Belgrade, Serbia). All other chemicals and solvents were of analytical grade and purchased from common sources. Water was treated in a Milli-Q water purification system (TGI Pure Water Systems, Greenville, SC, USA).

### 2.2. Plant Material

Seeds of six wild edible species were sown in sowing trays containing peat and they were transplanted in 2 L pots with peat and perlite in a ratio of 1:1; *v*/*v*. The seeds were collected from the wild by our team and were part of the seed collection of the Laboratory of Vegetable Production, University of Thessaly, Greece. The studied species included *Cichorium spinosum* L. (S1); *Centaurea raphanina* subsp. *mixta* (DC.) Runemark (S2); *Picris echioides* (L.) Holub (S3); *Urospermum picroides* (L.) Scop. ex. F.W. Schmidt (S4); *Sonchus oleraceus* L. (S5); and *S. asper* L. (S6). The cultivation took place in the winter–spring period of 2021, while the cultivation protocols followed a previously detailed procedure [21]. The leaves of each species were collected when they reached a size comparable to that of the plants handpicked in the wild (e.g., the rosette of leaves increased in size comprising green and tender leaves). After harvest, leaf samples were prepared by removing yellow and withered leaves, cleansing with distilled water, and drying with absorbent paper. Then, samples of separate leaves were placed in plastic bags in vacuum, kept under deep-freezing temperatures until they freeze-dried, and then stored under deep-freezing conditions (−80 °C) until extraction.

### 2.3. Hydroethanolic Extracts and Decoction Preparations

For each species, two samples were prepared. One sample included intact leaves that were used for the determination of the chemical profile and bioactive properties of raw leaves. Hydroethanolic extracts were obtained to evaluate the bioactive properties of raw leaves according to the protocol of Spréa et al. [39]. Briefly, 3 g of each sample was twice suspended in 80 mL of ethanol/water (80:20, *v*/*v*) and stirred at 150 rpm for 1 h at room temperature. When extraction was completed, the suspensions were passed through a Whatman No. 4 paper filter, the ethanol was removed with a rotary evaporator (Büchi R-2010, Flawil, Switzerland), and the aqueous fractions were frozen and lyophilized.

The other sample was used for the decoction preparation. Briefly, 3 g of plant material was used with the addition of 100 mL of boiling distilled water for 5 min. Then, the samples were left to cool down for 5 min and passed through a Whatman No. 4 paper filter. After decoction, leaf residues and the aqueous phase were stored in deep-freezing conditions and were lyophilized. After this, the leaf residues were used to evaluate their chemical profile, and the hydroethanolic extract was also prepared based on the method mentioned above.

### 2.4. Chemical Characterization

#### 2.4.1. Free Sugar and Organic Acid Composition

Free sugar content was determined by high-performance liquid chromatography coupled to a refraction index detector (HPLC-RI) (HPLC-RI, Knauer, Smartline 1000, and RI, Knauer, Berlin, Germany, respectively) using the previously described extracts, following the methodology of Spréa et al. [39]. The detected compounds were identified after comparison of relative retention times (Rts) with standard compounds, while quantification was implemented using melezitose (internal standard; IS). The processing of results was performed with Clarity 2.4 software (DataApex, Podohradska, Czech Republic). The results were expressed as g/100 g dry weight (dw).

Organic acid composition was assessed according to the protocol of Pereira et al. [40] using ultra-fast liquid chromatography coupled to a photodiode array detector (UFLC-PDA; Shimadzu Corporation, Kyoto, Japan) and a C18 SphereClone (Phenomenex, Torrance, CA, USA) reverse-phase column (5 µm, 250 × 4.6 mm i.d.). Chromatographic conditions and the identification and quantification procedure were described in detail in the work of Pereira et al. [39]. The results were expressed as g/100 g dw.

#### 2.4.2. Fatty Acid Profile and Tocopherol Composition

Fatty acid methyl ester (FAME) content was determined according to the method of Petropoulos et al. [38]. The detected compounds were identified and quantified using commercial standards and the obtained results were processed with Clarity DataApex 4.0 Software (Prague, Czech Republic). The content of fatty acids was expressed as the relative percentage of each fatty acid.

Tocopherol composition was also assessed according to the methodology and the equipment described in the work of Spréa et al. [38]. The detected compounds were identified using commercial standards, while quantification took place with the internal standard method using Tocol as the internal standard. The results were expressed as μg/100 g dw.

#### 2.4.3. Phenolic Compounds

The phenolic compounds were determined in the previously described extracts (see Section 2.2) after re-dissolving them in an ethanol/water solution (80:20, *v*/*v*) up to a final concentration of 10 mg/mL. For the analysis, the protocol and equipment used were described by Bessada et al. [41]. The detected compounds were identified and quantified based on the information of chromatographic behavior, spectra, and UV-vis masses, as well as after comparison with the available standard and literature data. The results were expressed as mg/g of extract.

### 2.5. Bioactive Properties

#### 2.5.1. Antioxidant Activity

The antioxidant activity was measured in the already described extracts via lipid peroxidation inhibition by thiobarbituric acid reactive substances (TBARSs) and oxidative hemolysis inhibition (OxHLIA) assays, following the protocol of Spréa et al. [38]. Trolox was used as a positive control. The results were expressed as the extract concentration that maintained 50% of the erythrocyte population intact (IC_50_, µg/mL) after Δt of 60 and 120 min.

#### 2.5.2. Antiproliferative Activity

Antiproliferative activity was determined in three human tumor cell lines, namely, CaCo2 (colorectal adenocarcinoma), NCI-H460 (non-small-cell lung cancer), and MCF-7 (breast adenocarcinoma), and a non-tumor cell line (PLP2, porcine liver primary cell culture, which was prepared from a freshly harvested porcine liver obtained from a local slaughter house), following the methodology cited by Spréa et al. [38]. Ellipticine was used as a positive control. Results were expressed as extract concentration responsible for 50% of cell growth inhibition (GI_50_, µg/mL).

#### 2.5.3. Anti-Inflammatory Activity

The anti-inflammatory activity of the extracts was determined in a lipopolysaccharide (LPS)-stimulated murine macrophage cell line (RAW 264.7), using the previously published protocols [42]. Dexamethasone was used as a positive control. Results were presented as the extract concentration that causes 50% NO production inhibition (EC_50_, µg/mL).

#### 2.5.4. Antimicrobial Activity

For the antimicrobial activity of the extracts, the Gram-positive bacteria *Staphylococcus aureus* (American Type Culture Collection, Manassas, VA, USA, ATCC 6538), *Bacillus cereus* (food isolate), and *Listeria monocytogenes* (National Collection of Type Cultures, London, UK, NCTC 7973), as well as the Gram-negative bacteria *Escherichia coli* (ATCC 25922), *Salmonella typhimurium* (ATCC 13311), and *Enterobacter cloacae* (ATCC 35030), were used. For antifungal assays, the following micromycetes were used: *Aspergillus ochraceus* (ATCC 12066), *A. niger* (ATCC 6275), *A. versicolor* (ATCC 11730), *Penicillium funiculosum* (ATCC 36839), *P. aurantiogriseum* (food isolate), and *Trichoderma viride* (IAM 5061). All the antimicrobial properties were assessed using the microdilution method [43]. The organisms were obtained from the Mycological Laboratory, Department of Plant Physiology, Institute for Biological Research ‘‘Siniša Stanković’’, Belgrade, Serbia. The food preservatives sodium benzoate (E211) and potassium metabisulfite (E224) were used as positive controls. The results were expressed as minimal inhibitory (MIC), bactericidal (MBC), or fungicidal (MFC) concentrations (mg/mL).

### 2.6. Statistical Analysis

The experiment was carried out according to a completely randomized design (CRD) with three replications per treatment. The results were expressed as mean values and standard deviations (SDs). Prior to analysis, data were checked to ensure that they followed normal distribution using the Shapiro–Wilk test and then analyzed with one-way analysis of variance (ANOVA) using Student’s *t*-test (*p* = 0.05) and Tukey’s HSD test (*p* = 0.05) when two or more means were compared, respectively. The statistical software used was JMP v. 16.1 (SAS Institute Inc., Cary, NC, USA).

## 3. Results and Discussion

### 3.1. Hydrophilic Compounds

Four sugars were identified in all the samples, namely, sucrose, glucose, fructose, and trehalose, although the latter was not detected in leaves after decoction (Table 1). Sucrose was the major sugar detected in the raw leaves of *Cichorium spinosum*, *Picris echioides*, *Sonchus oleraceus*, and *S. asper*, while fructose and glucose were the major free sugars in the raw leaves of *Centaurea raphanina* subsp. *mixta* and *Urospermum picroides* samples, respectively. Similar results were recorded for the leaves after decoction, except for the case of *C. spinosum,* where glucose was detected in higher amounts than sucrose. Another finding to be noted was the low content of free sugars in *C. raphanina* subsp. *mixta* leaves in both raw form and after decoction, which corroborates their extremely bitter taste, as these leaves had the lowest content sugar content. Thehalose was the least abundant free sugar with amounts that ranged between 0.44 g/100 g dw in *U. picroides* leaves and 0.93 g/100 g dw in *S. oleraceus* leaves. A similar profile of free sugars was reported for *C. raphanina* subsp. mixta and *C. spinosum* [21,44], while literature reports suggested that agronomic practices may affect the composition of free sugars [45,46]. Moreover, a significant variability in chemical profile can be observed among different ecotypes of the same species or between cultivated and wild plants [18,21]. Moreover, in all the studied species, raw leaves had a higher content of individual and total sugars than leaves after decoction, thus indicating a significant impact of this particular domestic processing on the chemical profile of leaves. Based on the finding of the work of Pinela et al. [47], the processing method may impact the profile of free sugars since disaccharides (e.g., sucrose and trehalose) are hydrolyzed in monosaccharides (e.g., fructose and glucose). Moreover, Andersson et al. [48] suggested that thermal processing results in the solubilization and leakage of sugars in the boiling water, a finding which justifies the reduction in leaves after decoction compared to the raw leaves observed in the current study.

The organic acids determined in this study were ascorbic, citric, malic, oxalic, quinic, shikimic, and fumaric acids, as presented in Table 2. From all the detected organic acids, oxalic acid was the richest one of all the leaf samples, either in raw form or after decoction, except for *C. raphanina* subsp. *mixta,* where citric acid was the richest compound. Previously, Petropoulos et al. [49] mentioned that oxalic acid, quinic acid, and malic acid were identified in *C. spinosum* leaves in descending order, while growth stage and fertilization regime may affect organic acid content and profile. Citric acid was also identified in higher amounts in wild and domesticated plants of *C. raphanina* subsp. *mixta* by Petropoulos et al. [21]. Oxalic acid was also mentioned as the most abundant organic acid in *S. oleraceus* plants, followed by malic acid and shikimic, ascorbic, citric, and fumaric acid, which were present in lower concentrations [50], while Petropoulos et al. [51] also indicated oxalic and malic acid as the main compounds in *P. echioides* and *U. picroides*.

Raw leaves and leaves after decoction presented the same profile of organic acids, although decoction resulted in a significant reduction in discrete and total organic acids for all the tested species. Organic acids, such as malic, quinic, oxalic, and citric acid, are often extracted in aqueous extracts, although decoctions are not usually rich in organic acids [52]. Oxalic acid is undesirable when consumed in high amounts, since it diminishes calcium bioavailability [53]. Guil et al. [54,55] have also highlighted the presence of toxic and antinutritional compounds in wild edible species, an aspect which has to be considered before suggesting the integration/introduction of these species in human diets. Considering that, in all the studied species, oxalic acid was the richest organic acid, cooking or processing with boiling water seems to be a beneficial method to reduce the content of this antinutritional factor. A similar trend has been reported in various wild edible greens, where various domestic cooking methods resulted in reduced nitrate content, which is also considered an undesired food component.

### 3.2. Lipophilic Compounds

The lipophilic compounds found in the samples (e.g., fatty acids and tocopherols) are cited in Table 3. In all the plant species, 22 fatty acids were detected with significant differences between the wild plant species and the composition before and after decoction. The richest compounds were α-linolenic acid (C18:3n3), linoleic acid (C18:2n6), and palmitic acid (C16:0), ranging from 35.8% to 53.8%, 9.44% to 24.14%, and 17.8% to 26.32%, respectively. Depending on plant species, myristic acid (C14:0), stearic acid (C18:0), behenic acid (C22:0), and lignoceric acid (C24:0) followed in ranging proportions, while the rest of the compounds were detected in values lower than 1%. *Picris echioides* had the highest content of α-linolenic acid (C18:3n3) and also the highest value of PUFAs in leaves before and after decoction. Moreover, all the wild species had a high level of PUFAs and a low level of SFAs, indicating their high nutritional value due to high ratios of PUFA/SFA. The lowest ratio of PUFAs/SFAs (1.31 and 1.24 in leaves before and after decoction, respectively) was recorded in *U. picroides* samples; however, even in this case, the ratio of PUFAs/SFAs was greater than 0.45, which is associated with beneficial health effects [33]. According to literature reports, wild edible plants have a high PUFA/SFA ratio since they consist mainly of α-linolenic acid (C18:3n3) followed by linoleic acid (C18:2n6) and palmitic acid (C16:0) [56,57]. However, fatty acid composition is highly dependent on the species, the ecotype, the developmental stage, and the cultivation practices, which may have a significant on fatty acid biosynthesis [46,58].

Decoction had a varied impact on fatty acid composition, especially on the major ones. In particular, α-linolenic acid showed a slight decrease in the leaves of all the species after decoction, while a similar trend was recorded for linoleic acid, apart from in the case of *C. spinosum* and *S. oleraceus,* where no effects were recorded. On the other hand, palmitic acid content increased after decoction for all the studied species. Similarly, SFAs increased after decoction for all the studied species, whereas MUFAs and PUFAs decreased (except for the MUFA of *S. asper,* where no significant changes were recorded). According to the literature, C18:3 fatty acids are synthesized through lipase activity which catalyzes the catabolism of lipids [59]. However, the activity of this enzyme is reduced under thermal processing [60], hence the decrease in α-linolenic acid content in the leaves after decoction in most of the species in our work (except for *C. raphanina* subsp. *mixta* where no differences were recorded). Fatty acid composition and *n* − 3 fatty acids are very significant for the nutritional value of wild edible plants [61]. Therefore, the impact of domestic cooking on the quality of the edible product should be considered for the adoption of healthy diets.

Regarding tocopherol content, α-tocopherol prevailed in all the plant species except for *C. spinosum,* where β-tocopherol was the most prevalent isoform of vitamin E, while no other tocopherols were detected (Table 3). Similarly, Morales et al. (2014) assessed different wild edible plants and suggested that α-tocopherol was the richest compound in the leaf samples, while γ-tocopherol was detected in lower amounts. De Paula Filho et al. [30] also recorded a varied tocopherol composition among three *Sonchus* species, with α-tocopherol being the most prevalent compound. In contrast to our study, the same authors [30] and Petropoulos et al. [21] detected γ-tocopherol in *Sonchus* sp. and *C. raphanina* subsp. *mixta*, while Petropoulos et al. [18] recorded significant amounts of δ-tocopherol in various *C. spinosum* ecotypes. However, the same authors [18] suggested that for certain genotypes, the cultivation practices may also affect tocopherol composition and content in wild edible species. This was also recorded in the work of Morales et al. [62] who identified all vitamin E isoforms in the basal leaves of *S. oleraceus* instead of only α-tocopherol.

Tocopherol content was affected by the decoction process since its values were lower in leaves after decoction in all the examined species, while total tocopherol content decreased from 25.3% (*C. raphanina* subsp. *mixta*) to 63.1% (*S. asper*). Tocopherols usually are not extracted to a high extent in aqueous decoctions owing to their lipophilic nature and low stability under thermal processing [63]. Therefore, their reduction in the leaves after decoction might be owing to their thermal degradation. In contrast, Kim et al. [64] reported that thermal processing may increase vitamin E content, depending on the food type and the processing method, since heat treatment may result in great losses of water-soluble components due to softening of cell tissues, thus having a concentration effect on the remaining components in food matrices.

### 3.3. Phenolic Compounds

The content of polyphenols in the hydroethanolic extracts of raw leaves, aqueous extracts obtained by decoction (decoction water), and the hydroethanolic extract of leaves after decoction for the examined species are presented in Table 4, Table 5, Table 6, Table 7, Table 8 and Table 9.

In *C. spinosum* leaf extracts, twenty-four individual phenolic compounds were identified, and total flavonoids were the richest class of polyphenols, regardless of the extraction method (Table 4). In particular, apigenin-*O*-acetylhexoside (peak 9; 35 mg/g of extract) was the richest compound in the hydroethanolic extract, followed by luteolin-O-hexoside-O-glucuronide (peak 10; 21 mg/g of extract) and cis-5-*O*-caffeoylquinic acid (peak 6; 15.2 mg/g of extract). The rest of the phenolic compounds were detected in lower amounts (<5 mg/g of extract). In general, hydroethanolic leaf extracts had significantly higher levels of individual and total phenolic compounds than decoctions and hydroethanolic extracts of leaves after decoction, except for *trans*-chicoric acid, where the highest amounts were identified in the hydroethanolic extracts of leaves after decoction. Moreover, apigenin-*O*-acetylhexoside (peak 9), which was the most abundant in the hydroethanolic extract, was not detected in decoctions, while it was detected in very small amounts (0.577 mg/g of extract) in leaves after decoction. Similarly, *trans*-5-*O*-caffeoylquinic acid (peak 6) and quercetin 4′-*O*-β-*D*-glucuronide (peak 17) were detected only in leaves after decoction (1.08 and 1.39 mg/g of extract, respectively), while *cis*-chicoric acid (peak 11) was identified only in decoctions (0.070 mg/g of extract).

Apigenin-O-acetylhexoside and luteolin-O-hexoside-O-glucuronide have not been mentioned before in hydromethanolic or aqueous extracts of *C. spinosum* leaves, while literature reports suggest chicoric acid as the main phenolic compound [58,65,66], which was detected only in decoctions in the present study. Moreover, the abovementioned studies had a lower content of total phenolic compounds than the hydroethanolic extract in our study, ranging from 7.20 to 23.5 mg/g of extract. *Trans*-5-O-Caffeoylquinic acid (peak 6), *cis*-chicoric acid (peak 11), quercetin 4′-O-β-D-glucuronide (peak 17), and kaempferol-3-O-glucuronide (peak 18) were only detected in decoctions and the hydroethanolic leaf extract after decoction. Moreover, in these treatments, kaempferol-3-O-glucuronide and *trans*-5-O-caffeoylquinic acid reached the highest content, respectively. In a previous study, Polyzos et al. [67] evaluated the phenolic compounds in hydroethanolic and aqueous extracts of this plant species and suggested 4-O-p-coumaroylquinic acid and isorhamnetin-O-hexuronoside as the main compounds, respectively, although fertilization regime had a significant impact on the bioactive compound content. The total phenolic compound content ranged from 4.68 to 5.34 mg/g of extract in hydroethanolic extracts and from 2.52 to 4.62 mg/g of extract in aqueous extracts, suggesting similar values with the decoctions of this study. Therefore, it has to be noted that the content of phenolic compounds in decoctions and in the leaves after decoction was very low compared to the extracts of raw leaves, a finding which suggests the severe effect of boiling on phenolic compounds. Similar findings were suggested by Sergio et al. [22] who tested the impact of various cooking methods (e.g., boiling, steaming, and microwaving) on the content of total phenolic compounds of various wild edible greens and reported a negative impact of cooking depending on the method and the species. The same findings were suggested by Miglio et al. [68] for carrots, broccoli florets, and courgettes, although the authors identified a varied impact depending on the species and the individual compound.

The results for *C. raphanina* subsp. *mixta* extracts were similar, where the highest values of discrete and total phenolic compounds were suggested in the hydroethanolic leaf extract, while in all the samples, total flavonoids was the richest class of compounds, accounting for more than 97% of total phenolic compounds (Table 5). Moreover, the highest amounts of kaempferol dimethylether hexoside (peak 1) and pinocembrin-*O*-arabirosyl-glucoside (peak 10) were detected in decoctions and in the leaves after decoction (0.73 and 1.51 mg/g of extract, respectively). Pinocembrin-*O*-acetylneohesperidoside isomer II (peak 14) and pinocembrin-*O*-neohesperidoside (peak 11) were the richest compounds in the hydroethanolic leaf extracts, reaching 28.7 and 22.8 mg/g of extract, respectively. Petropoulos et al. [21] also suggested the same major compounds in wild and cultivated plants of the species, although they recorded lower amounts compared to our study. Pinocembrin is not a very common flavanone and can be found not only in various plants but also in honey and propolis [65]. These compounds were also the richest in the leaf extracts after decoction with values of 11.9 and 9.8 mg/g of extract, respectively. Moreover, the decoctions recorded a different profile, with several compounds being identified in amounts between 0.501 and 0.78 mg/g of extract. According to the literature, apart from the cooking method [22,68], agronomic practices may have an impact on the phenolic compound composition of raw leaves of wild edible species [21,45,46].

Similarly to our work, Sergio et al. [22] identified a luteolin derivative as one of the main phenolic compounds of methanolic leaf extract, although chicoric acid recorded the highest content, which was also the case in decoctions in our study. Moreover, Petropoulos et al. [51] also reported that luteolin-*O*-glucuronide was the most abundant compound in methanolic extracts of leaves and its content can be affected by the growing period. These findings indicate that the extraction and processing method may affect both the yield of extraction and the profile of individual phenolic compounds.

For *U. picroides* extracts, significant differences were recorded among the tested extracts (Table 7). In this case, decoctions had the highest extraction yield of phenolic compounds (15.8 mg/g of extract of total phenolic compounds), followed by hydroethanolic extracts before and after decoction (10.46 and 9.3 mg/g of extract, respectively). Total flavonoids were the richest class of polyphenols in hydroethanolic extracts of leaves before and after decoction (9.27 and 6.5 mg/g of extract; and 89% and 70% of total phenolic compounds, respectively), while hydrolysable tannins were detected only in the hydroethanolic extracts of leaves after decoction (1.27 mg/g of extract). On the other hand, phenolic acids were the main class of phenolic compounds in decoctions, accounting for 66% of total phenolic compounds. Regarding the individual compounds, *cis*- and *trans*-5-O-caffeoylquinic acids were the richest compound in decoctions (5.3 and 4.0 mg/g of extract), while of the hydroethanolic extracts (before and after decoction), luteolin-6,8-di-*C*-hexoside was the richest compound (1.34 and 1.3 mg/g of extract, respectively).

In contrast to our study, Sergio et al. [22] indicated that chlorogenic acid was the major compound in *U. picroides*, while they also detected significant amounts of quercetin derivatives and di-caffeoylquinic acid. Moreover, Saber et al. [69], who performed a detailed metabolite profiling of the species, suggested the presence of several sesquiterpenes and sesquiterpenes lactones, as well as flavonoids and chlorogenic acids, while Petropoulos et al. [51] also detected most of the compounds identified in our study. However, none of the abovementioned studies identified luteolin, apigenin, or ellagic acid derivatives and oleuropein glucoside, which could be due to different extraction protocols, the genotype, or the growing conditions.

Finally, the two studied *Sonchus* species showed a varied profile of phenolic compounds in all the tested extracts with 14 and 13 compounds being identified in total in *S. oleraceus* and *S. asper*, respectively (Table 8 and Table 9). In *S. oleraceus* extracts, flavonoids was the prevailing class of phenolic compound in all the extracts with values that ranged between 90% (decoctions) and 95% (hydroethanolic extracts before and after decoction) of total phenolic compounds. Apigenin-*O*-glucuronide (peaks 12–14) was the richest compound in all the extracts with amounts that reached 46.2 mg/g, 2.8 mg/g, and 3.92 mg/g of extract in hydroethanolic extracts before decoction, in decoctions, and in hydroethanolic extracts after decoction, respectively. A similar profile was recorded for *S. asper* extracts, where total flavonoids was also the prevailing class of phenolic compound, accounting for 90%, 70%, and 97% of total phenolic compounds in hydroethanolic extracts before decoction, in decoctions, and in hydroethanolic extracts after decoction, respectively. Apigenin-O-glucuronide (peak 12) was also the richest compound, especially in the hydroethanolic extracts where it accounted for 50% and 76% of total phenolic compounds, before and after decoction, respectively. Despite the similarities, only seven of the identified compounds were common in the extracts of both species, with significant amounts of myricetin and kaempferol derivatives being detected in *S. oleraceus* extracts and (epi)catechin derivatives in the case of *S. asper.* In both species, decoctions resulted in the lowest yield of extraction, while the obtained amounts for decoctions were in the same range as in hydroethanolic extracts of leaves after decoction.

In a previous study, Aissani et al. [70] highlighted the importance of extraction solvent in phenolic compound content on *S. oleraceus* and suggested that hydromethanolic extracts contained more compounds than the aqueous ones. Similarly to our study, Juhaimi et al. [71] reported a high content of total flavonoids in hydromethanolic extracts of *S. oleraceus* young leaves, while they suggested gallic acid as the major compound. Petropoulos et al. [51] also detected high amounts of luteolin and apigenin derivatives, which accounted for 91% of the total phenolic compounds identified in hydromethanolic extracts of *S. oleraceus* leaves, while similar findings were reported by Gatto et al. [72] in hydromethanolic extracts of both *Sonchus* species. However, in the latter study, the major phenolic compounds were chicoric, caffeic, and chlorogenic acids that were not identified in this study. Stagos et al. [73] also suggested that chicoric acid was the most abundant compound in aqueous extracts of *S. asper,* followed by luteolin, apigenin, and caffeic acid, a difference that could be due to differences in the extraction protocol. Finally, to the best of our knowledge, myricetin-O-(O-galloyl)-hexoside, kaempferol-O-hexoside-O-hexoside, and eriodictyol-hexoside isomer were detected for the first time in *S. oleraceus* leaves.

Summarizing, there were significant differences between the extraction methods, with hydroethanolic showing greater amounts of phenolic compounds than decoctions in most of the studied species (except for *U. picroides* samples, where decoctions led to higher values of phenolic compounds). Similarly, Dia et al. [63,74] also reported that methanolic extracts of *Achillea millefolium* and *Taraxacum* sect. Ruderalia had a significantly higher total phenolic compound content than infusions and decoctions. These differences could be related not only to the extraction protocol (e.g., thermal processing may result in phenolic compound degradation [75]) but also the physical properties of the leaves of the studied species (e.g., texture, thickness of cuticle and epidermis, cell wall thickness, etc.), which may affect the extractability of phytochemicals from leaf tissues [76].

### 3.4. Bioactive Properties

The antioxidant properties of the tested species were evaluated with the TBARS and OxHLIA assays, as presented in Table 10. In both assays, hydroethanolic extracts obtained from raw leaves recorded the highest antioxidant potential for the studied species, followed by decoctions and the hydroethanolic extracts after decoction. Moreover, the latter extract obtained from *C. raphanina* subsp. *mixta* showed no activity for the OxHLIA assay at 60 and 120 min, while no significant differences were recorded for decoctions and hydromethanolic extracts of leaves after decoction for *P. echioides* and *U. picroides* for the OxHLIA assay at 60 min, as well as for *U. picroides* at 120 min. Finally, the hydroethanolic extracts of raw leaves of *S. oleraceus* recorded the lowest antioxidant potency, especially for the OxHLIA assay at 60 min, where IC_50_ values were similar to Trolox (19 and 19.6 μg/mL for the extract and Trolox, respectively).

Similarly, Guimarães et al. [77] reported higher antioxidant activity for the methanolic extracts of *Matricaria recutita* compared to decoctions through the TBARS assay, whereas the opposite trend was suggested for DPPH and reducing power assays due to varied mechanisms involved in the assessment of antioxidant activity. Moreover, hydromethanolic extracts of *Salvia officinalis* presented higher antioxidant activity than decoction due to higher contents of particular phenolic compounds [78]. Moreover, Polyzos et al. [67] also suggested that *C. spinosum* leaf extracts exerted higher antioxidant potency for the OxHLIA assay compared to the TBARS for the various fertilization regimes tested. Similarly to our work, Sergio et al. [22], who assessed the impact of various cooking methods on the antioxidant properties of various wild edible plants, also observed that *S. oleraceus* had higher antioxidant activity than *P. echioides* for all the tested cooking methods (e.g., boiling, steaming, microwaving), except for boiling, where no differences were recorded, whereas *U. picroides* showed higher activity than both of them for all the cooking methods.

Finally, Xia et al. [79], who tested six *Sonchus* species, reported that *S. oleraceus* extracts had higher activity than *S. asper* for four different assays (DPPH, ABTS, TBARS, and reducing power) and associated these properties with the higher content of phenolic compounds detected. This was also the case in our study for the hydroethanolic extracts of raw leaves, which had a higher content of total phenolic compounds than the other two extracts. However, it has to be noted that although decoctions of *U. picroides* samples contained more phenolic compounds than the other two extracts, this was not associated with higher antioxidant activity. Likewise, the highest overall content of total phenolic compounds for the hydroethanolic extracts of raw leaves of *C. spinosum* did not result in higher antioxidant activity than the rest of the species. Therefore, in addition to phenolic compounds, other bioactive compounds may present antioxidant capacity, as indicated by Petropoulos et al. [66], who reported low correlation coefficients for antioxidant activity and the content of phenolic compounds for various *C. spinosum* ecotypes.

The results of cytotoxic effects are presented in Table 10, with a varied response among the tested extracts against the various tumor and non-tumor cell lines. For CaCo2 and MCF-7 cells, the hydroethanolic extract of leaves after decoction had the highest activity, with no significant differences for the extracts obtained before and after decoction in the case of CaCo2 and MCF-7 cells for the samples of *C. spinosum* and *C. raphanina* subsp. *mixta*, respectively. On the other hand, hydroethanolic extracts before decoction were the most effective against NCI-H460 cells for all the studies species, apart from the case of *U. picroides,* where the extracts after decoction had the highest potency. For the anti-inflammatory activity, a varied response was recorded among the species, where the highest toxicity was recorded for the hydroethanolic extracts before decoction for the leaves of *C. spinosum* and *S. oleraceus*, the hydroethanolic extracts after decoction for *C. raphanina* subsp. *mixta*, *P. echioides,* and *S. asper*, and the decoctions for *U. picroides.* Finally, for the non-tumor cells (PLP2 cell line), the lowest activity was recorded for the decoctions for most of the species, except for *S. oleraceus* and *S. asper,* where the hydroethanolic extracts were less toxic. Regarding the individual species, the hydroethanolic extracts of raw leaves of *C. spinosum* had the highest efficacy against NCI-H460 and RAW 264.7 cells, while the lowest GI_50_ values for CaCo2 and MCF-7 cell lines were recorded for the hydroethanolic extracts after decoction of leaves of *C. raphanina* subsp. *mixta* and *P. echioides* (only for the MCF-7 cells).

The antiproliferative activity of *C. raphanina* subsp. *mixta* towards cancer cells has been indicated in previous studies, although the ecotype and cultivation techniques may affect this bioactivity [51,80]. Moreover, Alper and Güneş [14] reported the cytotoxic effects of ethanolic extracts of *U. picroides* flowering parts against various cancer cell lines (e.g., Daudi, A549 and HeLa), while the same extracts arrested the cycle of A549 and HeLa cells. On the other hand, Polyzos et al. [67] mentioned that *C. spinosum* hydroethanolic and aqueous extracts showed no cytotoxic effects towards the same cell lines tested in this work. Therefore, this difference could be due to the lower content of total phenolic compounds recorded in the study of Polyzos et al. [67] than the present study, or the differences in extracts’ composition, since specific phenolic compounds and their interactions are associated with cytotoxic effects [65,74].

### 3.5. Antimicrobial Activities

The antimicrobial effects of plant extracts are shown in Table 11. The results indicated high antibacterial and antifungal activity for all the tested plants with MIC and MBC values being lower than the positive controls implemented (E211 and E224). Regarding the antibacterial effects, all the species showed higher efficacy against *S. aureus* and *E. cloacae* compared to E211, as well as *B. cereus* compared to E224, while the MBC values of the studied extracts against the same bacterial strain were lower than those of E211. Overall, the studied extracts showed a varied response against *S. aureus* and *B. cereus*, while no differences were recorded for the rest of the bacterial strains tested.

Martins et al. [81] also observed that the decoctions and hydroethanolic extracts of *Origanum vulgare* had similar efficacy against a broad range of bacteria, indicating that the responsible compounds for the antibacterial properties are also water-soluble [82]. Petropoulos et al. [51] and Polyzos et al. [67] also suggested a varied antimicrobial effect for various wild edible greens, also suggesting differences due to the implemented cultivation practices. Moreover, Gatto et al. [72] also indicated significant antifungal properties for the extracts obtained from various wild edible herbs, including *S. oleraceus* and *S. asper*, while similar results were suggested by Antonia et al. [72], who evaluated the efficacy of extracts against postharvest fungal diseases. Finally, El-Desouky [83] recorded high activity against various *Aspergillus* species for the aqueous extracts of *S. oleraceus*.

## 4. Conclusions

Our results showed that the consumption of leaves has health-promoting properties owing to their bioactive phytochemical content, and they can be implemented as alternative ingredients in healthy diets. However, domestic cooking may have an impact on the chemical profile and bioactivities of the edible product. Therefore, although the raw leaves showed a higher nutritional value, the leaves after decoction showed a reduced content of oxalic acid, which is one of the main antinutritional factors detected in such species. Moreover, the extracts of raw leaves recorded a higher content of phenolic compounds for most of the species (except for *U. picroides*), which was associated with better antioxidant activity. Finally, the tested extracts showed varied cytotoxic and antimicrobial properties depending on the species and the extraction method. In conclusion, processing of wild edible species through cooking in boiling water does not severely affect the quality of the edible product, and the decoction water could find alternative uses in industrial sectors due to its antimicrobial and bioactive properties. However, further studies are needed with a larger number of wild edible plant species included.

## Figures and Tables

**Table 1:** Composition of free sugars (g/100 g dw) of leaf samples before and after decoctions (mean ± SD, *n* = 3).

Sample	Fructose	Glucose	Sucrose	Trehalose	Sum
Leaves					
*Cichorium spinosum*	1.09 ± 0.01 a	3.10 ± 0.05 a	3.17 ± 0.02 a	0.71 ± 0.02	8.1 ± 0.1 a
*Centaurea raphanina* subsp. *mixta*	1.34 ± 0.01 a	1.23 ± 0.04 a	1.02 ± 0.01 a	0.82 ± 0.02	4.42 ± 0.01 a
*Picris echioides*	1.95 ± 0.05 a	3.49 ± 0.07 a	2.48 ± 0.01 a	0.70 ± 0.01	8.6 ± 0.1 a
*Urospermum picroides*	2.01 ± 0.01 a	3.07 ± 0.06 a	3.58 ± 0.03 a	0.44 ± 0.01	9.1 ± 0.1 a
*Sonchus oleraceus*	1.60 ± 0.05 a	2.60 ± 0.01 a	3.69 ± 0.03 a	0.93 ± 0.01	8.8 ± 0.1 a
*Sonchus asper*	1.59 ± 0.05 a	2.47 ± 0.06 a	2.95 ± 0.01 a	0.84 ± 0.03	7.9 ± 0.1 a
Leaves after decoction					
*Cichorium spinosum*	0.83 ± 0.02 b	2.48 ± 0.03 b	2.06 ± 0.01 b	nd	5.36 ± 0.05 b
*Centaurea raphanina* subsp. *mixta*	1.14 ± 0.01 b	0.97 ± 0.01 b	0.85 ± 0.04 b	nd	2.96 ± 0.04 b
*Picris echioides*	1.48 ± 0.01 b	2.88 ± 0.01 b	1.73 ± 0.01 b	nd	6.10 ± 0.01 b
*Urospermum picroides*	1.66 ± 0.03 b	2.51 ± 0.01 b	2.72 ± 0.01 b	nd	6.89 ± 0.03 b
*Sonchus oleraceus*	1.18 ± 0.01 b	2.07 ± 0.02 b	2.71 ± 0.01 b	nd	5.95 ± 0.02 b
*Sonchus asper*	1.31 ± 0.01 b	1.79 ± 0.01 b	2.45 ± 0.06 b	nd	5.56 ± 0.06 b

Means in the same column and for the same sample followed by different Latin letters are significantly different at *p* < 0.05 according to Student’s *t*-test. nd—not detected.

**Table 2:** Composition of organic acids (g/100 g dw) of leaf samples before and after decoctions (mean ± SD, *n* = 3).

Sample	Oxalic Acid	Quinic Acid	Malic Acid	Ascorbic Acid	Shikimic Acid	Citric Acid	Fumaric Acid	Sum
	Leaves							
*Cichorium spinosum*	6.32 ± 0.03 a	5.01 ± 0.01	3.00 ± 0.02 a	tr	nd	tr	tr	14.32 ± 0.06 a
*Centaurea raphanina* subsp. *mixta*	0.977 ± 0.002 a	nd	2.52 ± 0.01 a	tr	nd	2.94 ± 0.03 a	tr	6.43 ± 0.03 a
*Picris echioides*	6.94 ± 0.04 a	nd	1.86 ± 0.02 a	nd	1.04 ± 0.01 a	nd	tr	9.84 ± 0.01 a
*Urospermum picroides*	5.80 ± 0.04 a	nd	2.96 ± 0.02 a	nd	0.794 ± 0.006 a	nd	tr	9.55 ± 0.06 a
*Sonchus oleraceus*	4.84 ± 0.01 a	nd	3.06 ± 0.02 a	nd	0.131 ± 0.002 a	nd	tr	8.04 ± 0.01 a
*Sonchus asper*	5.19 ± 0.06 a	nd	3.72 ± 0.07 a	nd	0.124 ± 0.001 a	nd	tr	9.04 ± 0.01 a
	Leaves after decoction							
*Cichorium spinosum*	5.19 ± 0.01 b	4.70 ± 0.02	2.00 ± 0.01 b	tr	nd	tr	tr	11.90 ± 0.01 b
*Centaurea raphanina* subsp. *mixta*	0.853 ± 0.002 b	nd	2.02 ± 0.01 b	tr	nd	2.45 ± 0.01 b	tr	5.33 ± 0.01 b
*Picris echioides*	5.96 ± 0.02 b	nd	1.19 ± 0.01 b	nd	0.824 ± 0.001 b	nd	tr	7.98 ± 0.02 b
*Urospermum picroides*	5.08 ± 0.01 b	nd	2.53 ± 0.01 b	nd	0.597 ± 0.001 b	nd	tr	8.21 ± 0.01 b
*Sonchus oleraceus*	4.60 ± 0.01 b	nd	2.89 ± 0.01 b	nd	0.063 ± 0.001 b	nd	tr	7.56 ± 0.01 b
*Sonchus asper*	4.49 ± 0.01 b	nd	3.03 ± 0.01 b	nd	0.047 ± 0.001 b	nd	tr	7.57 ± 0.01 b

nd—not detected; tr—traces. Means in the same column and for the same sample followed by different Latin letters are significantly different at *p* < 0.05 according to Student’s *t*-test.

**Table 3:** Lipophilic compounds in leaf samples before and after decoctions (mean ± SD, *n* = 3).

	Leaves						Leaves after Decoction					
Fatty Acids (%)	*Cichorium spinosum*	*Centaurea raphanina* subsp. *mixta*	*Picris echioides*	*Urospermum picroides*	*Sonchus oleraceus*	*Sonchus asper*	*Cichorium spinosum*	*Centaurea raphanina* subsp. *mixta*	*Picris echioides*	*Urospermum picroides*	*Sonchus oleraceus*	*Sonchus asper*
C6:0	0.24 ± 0.02 a	0.61 ± 0.04 b	0.34 ± 0.01 a	nd	0.63 ± 0.02 b	0.38 ± 0.01 a	0.183 ± 0.003 b	0.633 ± 0.003 a	0.325 ± 0.006 b	nd	0.677 ± 0.005 a	0.360 ± 0.001 b
C8:0	0.057 ± 0.003 b	0.056 ± 0.001 b	0.055 ± 0.004 b	nd	0.110 ± 0.003 b	0.088 ± 0.002 b	0.148 ± 0.003 a	0.085 ± 0.004 a	0.111 ± 0.002 a	nd	0.133 ± 0.001 a	0.957 ± 0.004 a
C10:0	0.055 ± 0.002 b	nd	0.097 ± 0.001 b	0.18 ± 0.01 b	0.066 ± 0.006 b	0.084 ± 0.001 b	0.205 ± 0.004 a	nd	0.13 ± 0.01 a	0.192 ± 0.001 a	0.102 ± 0.001 a	0.967 ± 0.004 a
C11:0	0.149 ± 0.003 a	0.141 ± 0.004 b	nd	0.118 ± 0.008 a	nd	nd	0.036 ± 0.001 b	0.162 ± 0.002 a	nd	0.122 ± 0.001 a	nd	nd
C12:0	0.048 ± 0.001 b	0.102 ± 0.001 b	0.095 ± 0.004 b	0.103 ± 0.006 b	0.21 ± 0.02 b	0.188 ± 0.002 a	0.308 ± 0.003 a	0.134 ± 0.004 a	0.117 ± 0.003 a	0.116 ± 0.001 a	0.224 ± 0.007 a	0.192 ± 0.004 a
C14:0	1.9 ± 0.1 a	0.508 ± 0.001 b	1.30 ± 0.03 b	0.86 ± 0.01 b	5.4 ± 0.3 b	1.4 ± 0.1 a	0.98 ± 0.01 b	0.554 ± 0.004 a	2.71 ± 0.01 a	0.905 ± 0.002 a	5.93 ± 0.01 a	1.45 ± 0.03 a
C15:0	0.26 ± 0.01 b	0.474 ± 0.008 a	0.222 ± 0.008 b	0.258 ± 0.008 b	0.21 ± 0.01 b	0.203 ± 0.009 b	0.695 ± 0.006 a	0.425 ± 0.007 b	0.240 ± 0.002 a	0.290 ± 0.001 a	0.317 ± 0.007 a	0.224 ± 0.007 a
C16:0	17.8 ± 0.5 b	25.5 ± 0.5 b	14.47 ± 0.01 b	23.3 ± 0.2 b	18.9 ± 0.2 b	17.85 ± 0.03 b	19.6 ± 0.2 a	26.32 ± 0.01 a	15.5 ± 0.1 a	23.96 ± 0.01 a	20.31 ± 0.05 a	17.96 ± 0.03 a
C16:1	2.46 ± 0.06 a	1.49 ± 0.01 a	1.4 ± 0.1 a	0.73 ± 0.07 a	2.33 ± 0.04 a	1.88 ± 0.02 a	1.81 ± 0.01 b	1.43± 0.01 b	0.949 ± 0.004 b	0.63 ± 0.01 b	2.22 ± 0.02 b	1.83 ± 0.02 b
C17:0	0.224 ± 0.006 b	0.47 ± 0.04 a	0.20 ± 0.01 b	0.296 ± 0.008 b	0.22 ± 0.02 b	0.241 ± 0.004 a	0.31 ± 0.01 a	0.453 ± 0.006 b	0.22 ± 0.004 a	0.346 ± 0.004 a	0.254 ± 0.001 a	0.245 ± 0.005 a
C18:0	1.72 ± 0.06 a	2.9 ± 0.1 a	1.83 ± 0.05 b	11.7 ± 0.2 a	2.32 ± 0.04 a	2.8 ± 0.2 a	1.73 ± 0.01 a	2.97 ± 0.01 a	2.23 ± 0.02 a	11.9 ± 0.1 a	2.34 ± 0.01 a	2.91 ± 0.01 a
C18:1n9c	2.25 ± 0.02 b	2.27 ± 0.03 a	5.2 ± 0.3 a	3.1 ± 0.1 a	2.28 ± 0.07 a	3.3 ± 0.1 a	2.32 ± 0.02 a	2.13 ± 0.01 b	5.14 ± 0.01 a	2.8 ± 0.1 b	2.21 ± 0.01 b	3.23 ± 0.07 a
C18:2n6c	17.8 ± 0.3 a	24.14 ± 0.08 a	15.3 ± 0.3 a	9.71 ± 0.05 a	10.0 ± 0.3 a	12.92 ± 0.04 a	17.7 ± 0.3 a	23.08 ± 0.04 b	14.12 ± 0.02 b	9.44 ± 0.02 b	10.05 ± 0.07 a	11.71 ± 0.04 b
C18:3n3	48.9 ± 0.4 a	35.8 ± 0.5 a	53.8 ± 0.4 a	44.3 ± 0.3 a	51.3 ± 0.5 a	52.5 ± 0.3 a	47.5 ± 0.1 b	35.81 ± 0.02 a	52.1 ± 0.1 b	43.5 ± 0.1 b	49.3 ± 0.1 b	51.8 ± 0.1 b
C20:0	0.52 ± 0.03 b	0.66 ± 0.04 b	1.22 ± 0.06 b	0.77 ± 0.02 b	1.09 ± 0.04 b	1.79 ± 0.08 a	0.57 ± 0.01 a	1.08 ± 0.06 a	1.34 ± 0.05 a	0.88 ± 0.01 a	1.17 ± 0.01 a	1.77 ± 0.03 a
C20:1	0.078 ± 0.003 a	0.207 ± 0.007 a	0.29 ± 0.02 a	nd	0.025 ± 0.001	0.029 ± 0.002	0.072 ± 0.001 b	0.208 ± 0.004 a	0.301 ± 0.001 a	nd	nd	nd
C20:2	0.163 ± 0.005 a	0.14 ± 0.01 a	0.137 ± 0.001 a	nd	0.080 ± 0.003	0.080 ± 0.004	0.107 ± 0.004 b	0.105 ± 0.001 b	0.122 ± 0.001 b	nd	nd	nd
C21:0	0.142 ± 0.001 b	0.211 ± 0.008 b	0.112 ± 0.004 b	0.204 ± 0.007 b	0.181 ± 0.002 a	0.15 ± 0.01 a	0.30 ± 0.01 a	0.22 ± 0.01 a	0.204 ± 0.001 a	0.273 ± 0.002 a	0.19 ± 0.01 a	0.154 ± 0.001 a
C22:0	1.20 ± 0.05 b	1.22 ± 0.01 b	0.81 ± 0.01 b	1.16 ± 0.01 b	2.8 ± 0.2 a	1.833 ± 0.001 b	1.45 ± 0.02 a	1.26 ± 0.03 a	0.914 ± 0.001 a	1.193 ± 0.001 a	2.71 ± 0.02 a	1.942 ± 0.001 a
C22:1	0.98 ± 0.02 a	0.98 ± 0.06 a	0.9 ± 0.1 a	0.98 ± 0.08 a	0.110 ± 0.002 a	0.92 ± 0.03 a	0.88 ± 0.01 b	0.97 ± 0.02 a	0.94 ± 0.01 a	0.91 ± 0.01 a	0.10 ± 0.01 a	0.939 ± 0.004 a
C23:0	0.73 ± 0.06 b	0.379 ± 0.004 b	0.24 ± 0.01 b	0.307 ± 0.007 b	0.29 ± 0.02 b	0.258 ± 0.002 a	0.81 ± 0.01 a	0.45 ± 0.01 a	0.382 ± 0.004 a	0.319 ± 0.005 a	0.325 ± 0.005 a	0.260 ± 0.001 a
C24:0	2.19 ± 0.09 b	1.5 ± 0.1 a	1.82 ± 0.01 b	1.9 ± 0.2 b	1.34 ± 0.03 b	1.16 ± 0.01 a	2.31 ± 0.01 a	1.52 ± 0.01 a	1.91 ± 0.03 a	2.16 ± 0.02 a	1.41 ± 0.02 a	1.08 ± 0.06 b
SFA	27.3 ± 0.1 b	34.8 ± 0.7 b	22.80 ± 0.05 b	41.21 ± 0.2 b	33.8 ± 0.2 b	28.3 ± 0.2 b	29.6 ± 0.2 a	36.27 ± 0.02 a	26.35 ± 0.07 a	42.68 ± 0.01 a	36.10 ± 0.09 a	30.46 ± 0.02 a
MUFA	5.77 ± 0.01 a	4.9 ± 0.01 a	7.9 ± 0.1 a	4.79 ± 0.02 a	4.75 ± 0.03 a	6.1 ± 0.2 a	5.1 ± 0.1 b	4.73 ± 0.03 b	7.34 ± 0.01 b	4.36 ± 0.01 b	4.54 ± 0.01 b	6.00 ± 0.08 a
PUFA	66.9 ± 0.1 a	60.3 ± 0.6 a	69.3 ± 0.1 a	54.0 ± 0.2 a	61.5 ± 0.3 a	65.5 ± 0.2 a	65.3 ± 0.3 b	59.00 ± 0.06 b	66.31 ± 0.05 b	52.96 ± 0.01 b	59.36 ± 0.08 b	63.5 ± 0.1 b
Tocopherols (µg/100 g dw)												
*α*-tocopherol	679 ± 5 a	418 ± 5 a	865 ± 5 a	201 ± 4 a	280 ± 4 a	223 ± 3 a	216 ± 5 b	333 ± 4 b	478 ± 3 b	112 ± 2 b	186.2 ± 0.3 b	117.2 ± 0.6 b
*β*-tocopherol	2712 ± 2 a	242 ± 2 a	143 ± 2	66.0 ± 0.1	177 ± 2	95 ± 2	1632 ± 8 b	160 ± 2 b	nd	nd	nd	nd
Sum	3391 ± 3 a	660 ± 3 a	1008 ± 3 a	267 ± 4 a	457 ± 2 a	318 ± 5 a	1848 ± 3 b	493 ± 2 b	478 ± 3 b	112 ± 2 b	186.2 ± 0.3 b	117.2 ± 0.6 b

nd—not detected. Means in the same row and for the same sample followed by different Latin letters are significantly different at *p* < 0.05 according to Student’s *t*-test.

**Table 4:** Retention time (Rt), wavelengths of maximum absorption in the visible region (λmax), mass spectral data, tentative identification, and quantification (mg/g of extract) of the phenolic compounds present in the hydroethanolic extracts of leaves, decoctions, and hydroethanolic extracts of leaves of *Cichorium spinosum* after decoction (mean ± SD, *n* = 3).

						Quantification		
Peak	Rt (min)	λmax (nm)	[M–H]^−^ (*m*/*z*)	MS^2^ (*m*/*z*)	Tentative Identification	Hydroethanolic Extracts	Decoctions	Hydroethanolic Extracts after Decoction
1	4.52	328	311	179 (85), 149 (54), 135 (100)	Caftaric acid	1.42 ± 0.07 a	0.190 ± 0.006 b	nd
2	4.85	284	341	179 (100)	Caffeic acid hexoside	2.20 ± 0.09 a	nd	0.077 ± 0.002 b
3	5.70	316	341	179 (100)	Caffeic acid hexoside isomer 1	1.13 ± 0.07 a	nd	0.053 ± 0.002 b
4	5.97	311	377	191 (90),173 (5),163 (100),155 (3),137 (5),119 (4)	*cis* 3-*p*-Coumarouylquinic acid	0.972 ± 0.002 a	0.10 ± 0.01 b	nd
5	6.59	324	353	191 (100),179 (4),161 (5),135 (3)	*cis*-5-*O*-Caffeoylquinic acid	15.2 ± 0.8 a	0.32 ± 0.02 b	0.27 ± 0.01 c
6	7.01	324	353	191 (100),179 (4),161 (5),135 (3)	*trans*-5-*O*-Caffeoylquinic acid	nd	nd	1.08 ± 0.05
7	9.63	336	593	503 (32),473 (100), 383 (12), 353 (22), 325 (11)	Apigenin 6,8-*C*-diglucoside	2.6 ± 0.1 a	0.300 ± 0.001 b	0.212 ± 0.002 c
8	12.08	273	321	169 (100)	Digallic acid	3.4 ± 0.2 a	nd	0.028 ± 0.001 b
9	13.05	335	473	269 (100)	Apigenin-*O*-acetylhexoside	35 ± 1 a	nd	0.577 ± 0.007 b
10	13.42	350	623	461 (100), 285 (26)	Luteolin-*O*-hexoside-*O*-glucuronide	21.0 ± 0.7 a	0.58 ± 0.02 c	0.76 ± 0.04 b
11	14.43	328	473	311 (100), 293 (92), 179 (10)	*cis*-Chicoric acid	nd	0.070 ± 0.004	nd
12	15.16	334	609	285 (100)	Kaempferol-*O*-hexoside-*O*-hexoside	1.17 ± 0.05 a	0.54 ± 0.01 b	1.13 ± 0.06 a
13	15.41	328	473	311 (95), 293 (100), 179 (8)	*trans*-Chicoric acid	0.461 ± 0.009 b	0.104 ± 0.002 c	0.511 ± 0.008 a
14	17.97	348	593	285 (100)	Kaempferol-3-*O*-rutinoside	0.69 ± 0.02 a	0.60 ± 0.01 c	0.659 ± 0.001 b
15	18.08	342	477	301 (100)	Quercetin-3-*O*-glucuronide	3.52 ± 0.04 a	nd	0.572 ± 0.003 b
16	18.57	344	477	301 (100)	Quecetin 3-*O*-β-*D*-glucuronide	2.32 ± 0.04 a	nd	1.56 ± 0.04 b
17	18.77	342	477	301 (100)	Quercetin 4′-*O*-β-*D*-glucuronide	nd	nd	1.39 ± 0.06
18	18.84	348	461	285 (100)	Kaempferol-3-*O*-glucuronide	nd	0.88 ± 0.04 a	0.76 ± 0.03 b
19	20.22	356	505	463 (10), 301 (100)	Quercetin-7-*O*-(6″-*O*-acetyl)glucoside 2	1.04 ± 0.01 a	0.58 ± 0.01 c	0.62 ± 0.01 b
20	21.01	343	593	285 (100)	Luteolin 7-*O*-glucoside	1.6 ± 0.1 a	nd	0.94 ± 0.07 b
21	21.91	343	461	285 (100)	Luteolin-glucuronide	4.7 ± 0.4 a	nd	2.6 ± 0.1 b
22	22.89	290	481	301 (95), 275 (24)	Mono-HHDP hexoside	1.89 ± 0.05	nd	nd
23	23.42	291	481	301 (98), 275 (21)	Mono-HHDP hexoside	2.24 ± 0.06 a	nd	1.98 ± 0.02 b
24	24.65	290	481	301 (92), 275 (18)	Mono-HHDP hexoside	1.53 ± 0.03 a	nd	1.42 ± 0.02 b
					TPA	24.8 ± 0.7 a	0.788 ± 0.01 c	2.015 ± 0.05 b
					TF	73 ± 3 a	3.48 ± 0.02 c	11.80 ± 0.01 b
					THT	5.67 ± 0.04 a	-	3.40 ± 0.01 b
					TPC	104 ± 2 a	4.26 ± 0.01 c	17.21 ± 0.06 b

nd: not detected; TPA: total phenolic acids; TF: total flavonoids; THT: total hydrolysable tannins; TPC: total phenolic compounds. Calibration curves used in the quantification were standard calibration curves: caffeic acid (y = 388,345x + 406,369, *R*^2^ = 0.999, limit of detection (LOD) = 0.78 µg/mL and limit of quantitation (LOQ) = 1.97 µg/mL, peaks 1, 2, 3, 11 and 13); *p*-coumaric acid (y = 301,950x + 6966.7, *R*^2^ = 0.9999, LOD = 0.68 µg/mL and LOQ = 1.61 µg/mL, peak 4); chlorogenic acid (y = 168,823x − 161,172, *R*^2^ = 0.999, LOD = 0.20 µg/mL and LOQ = 0.68 µg/mL, peaks 5 and 6); apigenin-6-*C*-glucoside (y = 107,025x + 61,531, *R*^2^ = 0.9989, LOD = 0.19 µg/mL and LOQ = 0.63 µg/mL, peak 7); gallic acid (y = 131,538x + 292,163, *R*^2^ = 0.9969, LOD = 8.05 µg/mL and LOQ = 24.41 µg/mL, peak 8); apigenina-7-*O*-glucósido (y = 10,683x − 45,794, *R*^2^ = 0.996, LOD = 136.95 µg/mL and LOQ = 414.98 µg/mL, peaks 9, 10, 20 and 21); quercetin-3-*O*-glucoside (y = 34,843x − 160,173, *R*^2^ = 0.9998, LOD = 0.21 µg/mL and LOQ = 0.71 µg/mL, peaks 12 and 14–19); and ellagic acid (y = 26,719x − 317,255, *R*^2^ = 0.9986, LOD = 41.20 µg/mL and LOQ = 124.84 µg/mL, peaks 22–24). Means in the same row followed by different Latin letters are significantly different at *p* < 0.05 according to Tukey’s HSD test. Significant differences (*p* < 0.001) between two samples were assessed by a Student’s *t*-test. Means in the same row and for the same sample followed by different Latin letters are significantly different at *p* < 0.05 according to Student’s *t*-test.

**Table 5:** Retention time (Rt), wavelengths of maximum absorption in the visible region (λmax), mass spectral data, tentative identification, and quantification (mg/g of extract) of the phenolic compounds present in the hydroethanolic extracts of leaves, decoctions, and hydroethanolic extracts of leaves of *Centaurea raphanina* subsp. *mixta* after decoction (mean ± SD, *n* = 3).

						Quantification		
Peak	Rt (min)	λmax (nm)	[M–H]^−^ (*m*/*z*)	MS^2^ (*m*/*z*)	Tentative Identification	Hydroethanolic Extracts	Decoctions	Hydroethanolic Extracts after Decoction
1	14.64	302	475	313(100)	Kaempferol dimethylether hexoside	nd	0.73 ± 0.03 a	0.676 ± 0.004 b
2	16.75	330	355	193(80), 179(100), 161(17)	Ferulic acid-*O*-hexoside	0.264 ± 0.003 a	0.21 ± 0.01 c	0.230 ± 0.009 b
3	17.35	326	581	461(100), 299(24)	Diosmetin-*C*-dihexoside	0.26 ± 0.02 a	tr	0.023 ± 0.001 b
4	18.66	334	461	285 (100)	Kaempherol-*O*-glucuronide	1.28 ± 0.05 a	0.596 ± 0.001 c	0.83 ± 0.01 b
5	20.11	334	579	285 (100)	Kaempherol-*O*-hexosyl-pentoside	1.42 ± 0.08 a	0.57 ± 0.02 c	0.90 ± 0.03 b
6	21.81	334	563	269 (100)	Apigenin-*O*-hexosyl-pentoside	2.0 ± 0.1 a	0.73 ± 0.02 c	1.20 ± 0.06 b
7	23.10	334	445	269 (100)	Apigenin-*O*-glucuronide	nd	0.603 ± 0.006 b	0.97 ± 0.06 a
8	25.33	332	665	621 (100), 285 (45)	Kaempherol-*O*-malonyl-pentoside	0.707 ± 0.001 a	0.528 ± 0.001 c	0.614 ± 0.002 b
9	26.90	334	605	545(33), 431(33), 311(27), 269(100)	Acetylated apigenin-*C*-hexoside-*O*-pentoside	1.14 ± 0.06 a	0.58 ± 0.01 c	0.723 ± 0.004 b
10	27.89	286/326	549	429 (12), 297 (14), 279 (5), 255 (41)	Pinocembrin-*O*-arabirosyl-glucoside	0.69 ± 0.02 b	0.530 ± 0.001 c	1.51 ± 0.04 a
11	29.14	286/326	563	443 (12), 401 (5), 297 (21), 255 (58)	Pinocembrin-*O*-neohesperidoside	22.8 ± 0.2 a	0.78 ± 0.02 c	11.9 ± 0.3 b
12	31.28	286/328	591	549 (30), 429 (20), 297 (15), 279 (5), 255 (32)	Pinocembrin-*O*-acetylarabirosyl-glucoside	5.0 ± 0.3 a	0.544 ± 0.003 c	1.40 ± 0.05 b
13	31.75	286/326	605	563 (12), 545 (5), 443 (30), 401 (10), 255 (40)	Pinocembrin-*O*-acetylneohesperidoside isomer I	5.4 ± 0.1 a	0.501 ± 0.006 c	3.3 ± 0.1 b
14	32.14	286/328	605	563 (10), 545 (5), 443 (28), 401 (9), 255 (39)	Pinocembrin-*O*-acetylneohesperidoside isomer II	28.7 ± 0.4 a	0.618 ± 0.001 c	9.8 ± 0.6 b
					TPA	0.264 ± 0.01 a	0.208 ± 0.01 c	0.230 ± 0.01 b
					TF	69.4 ± 0.3 a	7.09 ± 0.01 c	33.8 ± 0.4 b
					TPC	69.7 ± 0.3 a	7.30 ± 0.01 c	34.0 ± 0.4 b

nd: not detected; tr: traces; TPA: total phenolic acids; TF: total flavonoids; TPC: total phenolic compounds. Calibration curves used in the quantification were tandard calibration curves: quercetin-3-*O*-glucoside (y = 34,843x − 160,173, *R*^2^ = 0.9998, LOD = 0.21 µg/mL and LOQ = 0.71 µg/mL, peaks 1, 4, 5, 8 and 10–14); ferulic acid (y = 633,126x − 185,462, *R*^2^ = 0.999, LOD = 1.85 µg/mL and LOQ = 5.61 µg/mL, peak 2); naringenin (y = 18,433x + 78,903, *R*^2^ = 0.9998, LOD = 18.66 µg/mL and LOQ = 56.55 µg/mL, peak 3); and apigenina-7-*O*-glucósido (y = 10,683x − 45,794, *R*^2^ = 0.996, LOD = 136.95 µg/mL and LOQ = 414.98 µg/mL, peaks 6, 7 and 9). Means in the same row followed by different Latin letters are significantly different at *p* < 0.05 according to Tukey’s HSD test. Significant differences (*p* < 0.001) between two samples were assessed by Student’s *t*-test. In *P. echioides* extracts, twenty-three phenolic compounds were identified in total with significant differences between the extraction methods (Table 6). Hydroethanolic extracts had the highest total phenolic compound content, which comprised mostly phenolic acids (73% of total phenolic compounds), and the highest number of individual compounds identified (nineteen compounds). In contrast to *C. spinosum* and *C. raphanina* subsp. *mixta*, decoctions contained the second highest amount of total phenolic compounds, equally distributed to phenolic acids and flavonoids, while in both decoctions and in hydromethanolic extracts of leaves after decoction, only fourteen individual compounds were identified. Finally, total flavonoids was the prevailing class of phenolic compounds in hydromethanolic extracts of leaves after decoction accounting for 92.8% of total phenolic compounds. Luteolin-7-O-β -D-glucopyranoside (peak 21) was the richest compound in the hydroethanolic extract (5.0 mg/g of extract), followed by luteolin-7-O-β-D-glucopyranoside (peak 20; 3.1 mg/g of extract). In decoctions and hydroethanolic extracts of leaves after decoction, the most abundant compounds were *trans*-chicoric acid (peak 11; 2.02 mg/g of extract) and quercetin-3-*O*-glucuronide (peak 13; 1.50 mg/g of extract), respectively.

**Table 6:** Retention time (Rt), wavelengths of maximum absorption in the visible region (λmax), mass spectral data, tentative identification, and quantification (mg/g of extract) of the phenolic compounds present in the hydroethanolic extracts of leaves, decoctions, and hydroethanolic extracts of leaves of *Picris echioides* after decoction (mean ± SD, *n* = 3).

						Quantification		
Peak	Rt (min)	λmax (nm)	[M–H]^−^ (*m*/*z*)	MS^2^ (*m*/*z*)	Tentative Identification	Hydroethanolic Extracts	Decoctions	Hydroethanolic Extracts after Decoction
1	4.41	328	311	179 (85), 149 (54), 135 (100)	Caftaric acid	0.94 ± 0.05 a	0.67 ± 0.03 c	0.081 ± 0.004 b
2	5.48	316	341	179 (100)	Caffeic acid hexoside	0.48 ± 0.02 a	0.053 ± 0.003 b	0.014 ± 0.001 c
3	6.59	325	353	191 (100),179 (6),161 (5),135 (4)	*cis*-5-*O*-Caffeoylquinic acid	1.12 ± 0.05 a	0.56 ± 0.03 b	0.229 ± 0.008 c
4	6.95	324	353	191 (100),179 (4),161 (5),135 (3)	*trans*-5-*O*-Caffeoylquinic acid	1.18 ± 0.07 a	0.65 ± 0.04 b	0.24 ± 0.01 c
5	8.59	291	343	191 (100),169 (13)	Galloylquinic acid	0.060 ± 0.003 a	0.006 ± 0.001 b	nd
6	9.52	362	433	301 (100)	Ellagic acid-pentoside	1.30 ± 0.01 b	nd	1.42 ± 0.02 a
7	9.98	330	593	473 (6), 429 (51), 284 (80), 285 (40)	Kaempferol 3-*O*-(*O*-rhamnosyl)hexoside	0.74 ± 0.02 a	nd	0.525 ± 0.008 b
8	11.26	311	337	191 (100), 163 (23), 145 (7), 119 (5)	*trans*-5-*p*-Coumaroylquinic acid	1.06 ± 0.02 b	1.48 ± 0.09 a	nd
9	12.55	300sh328	473	311 (100), 293 (60), 179 (10)	Caffeoyl hexosylpentoside	1.00 ± 0.03	nd	nd
10	14.41	328	473	311 (100), 293 (90), 219 (5), 179 (10), 149 (3), 135 (3)	*cis*-Chicoric acid	0.64 ± 0.02 b	0.69 ± 0.03 a	tr
11	15.48	326	473	311 (100), 293 (90), 219 (5), 179 (10), 149 (3), 135 (3)	*trans*-Chicoric acid	0.52 ± 0.03 b	2.02 ± 0.06 a	tr
12	15.58	329	609	285 (100)	Luteolin-6,8-di-*C*-hexoside	2.01 ± 0.07 c	2.4 ± 0.1 b	2.49 ± 0.05 a
13	18.11	352	477	301 (100)	Quercetin-3-*O*-glucuronide	2.80 ± 0.07 a	0.97 ± 0.03 c	1.50 ± 0.05 b
14	19.97	350	549	505 (100), 463 (22), 301 (50)	Quercetin-*O*-malonylhexoside	2.35 ± 0.05 a	0.69 ± 0.02 b	0.70 ± 0.04 b
15	21.14	323	487	325 (100), 307 (57), 293 (85), 193 (30)	Feruloyl hexosylpentoside	0.55 ± 0.03 b	nd	0.130 ± 0.005 a
16	21.68	347	461	285 (100)	Kaempferol-*O*-glucuronide	nd	0.830 ± 0.004	nd
17	23.12	350	491	315 (100)	Isorhamnetin-*O*-glucuronide	nd	0.764 ± 0.009 a	0.560 ± 0.004 b
18	23.23	336	749	557, 541, 367, 353	Vicenin derivative	1.50 ± 0.02	nd	nd
19	24.40	347	533	489 (67), 285 (100)	Kaempferol-*O*-malonylhexoside	nd	0.61 ± 0.02 a	0.485 ± 0.005 b
20	27.67	340	489	285 (100)	Luteolin-7-*O*-β-*D*-glucopyranoside	3.1 ± 0.1 a	nd	0.75 ± 0.01 b
21	28.52	282	685	493 (100), 337 (21)	Luteolin-7-*O*-β-*D*-glucopyranoside	5.0 ± 0.1	nd	nd
22	31.56	329	609	563 (100), 285 (42)	Luteolin-6,8-di-*C*-hexoside	nd	nd	0.26 ± 0.06
23	32.57	344	649	607 (6), 431 (42), 285 (31)	Kaempferol (acyl)glucuronide-*O*-rhamnoside	1.69 ± 0.01	nd	nd
					TPA	7.5 ± 0.1 a	6.1 ± 0.1 b	0.669 ± 0.02 c
					TF	20.6 ± 0.1 a	6.3 ± 0.2 c	8.69 ± 0.03 b
					TPC	28.07 ± 0.04 a	12.42 ± 0.08 b	9.36 ± 0.05 c

nd: not detected; TPA: total phenolic acids; TF: total flavonoids; TPC: total phenolic compounds. Calibration curves used in the quantification were standard calibration curves: caffeic acid (y = 388,345x + 406,369, *R*^2^ = 0.999, limit of detection (LOD) = 0.78 µg/mL and limit of quantitation (LOQ) = 1.97 µg/mL, peaks 1, 2 and 9–11); chlorogenic acid (y = 168,823x − 161,172, *R*^2^ = 0.999, LOD = 0.20 µg/mL and LOQ = 0.68 µg/mL, peaks 3, 4 and 8); gallic acid (y = 131,538x + 292,163, *R*^2^ = 0.9969, LOD = 8.05 µg/mL and LOQ = 24.41 µg/mL, peak 5); ellagic acid (y = 26,719x − 317,255, *R*^2^ = 0.9986, LOD = 41.20 µg/mL and LOQ = 124.84 µg/mL, peak 6); quercetin-3-*O*-glucoside (y = 34,843x − 160,173, *R*^2^ = 0.9998, LOD = 0.21 µg/mL and LOQ = 0.71 µg/mL, peaks 7, 13, 14, 16–19 and 23); apigenin-6-*C*-glucoside (y = 107,025x + 61,531, *R*^2^ = 0.9989, LOD = 0.19 µg/mL and LOQ = 0.63 µg/mL, peaks 12 and 22); ferulic acid (y = 633,126x − 185,462, *R*^2^ = 0.999, LOD = 1.85 µg/mL and LOQ = 5.61 µg/mL, peak 15); and apigenina-7-*O*-glucósido (y = 10,683x − 45,794, *R*^2^ = 0.996, LOD = 136.95 µg/mL and LOQ = 414.98 µg/mL, peaks 20 and 21). Means in the same row followed by different Latin letters are significantly different at *p* < 0.05 according to Tukey’s HSD test. Significant differences (*p* < 0.001) between two samples were assessed by Student’s *t*-test.

**Table 7:** Retention time (Rt), wavelengths of maximum absorption in the visible region (λmax), mass spectral data, tentative identification, and quantification (mg/g of extract) of the phenolic compounds present in the hydroethanolic extracts of leaves, decoctions, and hydroethanolic extracts of leaves of *Urospermum picroides* after decoction (mean ± SD, *n* = 3).

						Quantification		
Peak	Rt (min)	λmax (nm)	[M−H]^−^ (*m*/*z*)	MS^2^ (*m*/*z*)	Tentative Identification	Hydroethanolic Extracts	Decoctions	Hydroethanolic Extracts after Decoction
1	6.46	325	353	191 (100), 179 (6), 161 (5), 135 (4)	*cis*-5-*O*-Caffeoylquinic acid	1.02 ± 0.01 b	5.3 ± 0.1 a	0.27 ± 0.01 c
2	6.99	324	353	191 (100), 179 (4), 161 (5), 135 (3)	*trans*-5-*O*-Caffeoylquinic acid	nd	4.0 ± 0.1 a	0.96 ± 0.06 b
3	9.15	362	433	301 (100)	Ellagic acid-pentoside	nd	nd	1.274 ± 0.002
4	11.19	311	337	191 (100), 163 (23), 145 (7), 119 (5)	*trans*-5-*p*-Coumaroylquinic acid	nd	0.441 ± 0.002 a	0.050 ± 0.001 b
5	12.72	288	705	529 (100), 337 (18), 191 (3), 161 (2)	3,7-*O*-diferuloyl-4-*O*-caffeoyl quinic acid	0.173 ± 0.003 c	0.79 ± 0.03 a	0.256 ± 0.005 b
6	14.71	335	431	385, 269 (100)	Apigenin-7-*O*-glucoside	1.01 ± 0.01 b	1.28 ± 0.02 a	0.62 ± 0.04 c
7	15.04	334	609	285 (100)	Kaempferol-*O*-hexoside-*O*-hexoside	nd	nd	0.544 ± 0.005 a
8	15.54	326	473	311 (100), 293 (90), 219 (5), 179 (10), 149 (3), 135 (3)	*trans*-Chicoric acid	nd	nd	tr
9	18.06	354	463	301 (100)	Quercetin-3-*O*-glucoside	0.579 ± 0.002 b	0.63 ± 0.03 a	0.583 ± 0.007 b
10	18.66	325	461	285 (100)	Kaempferol-*O*-glucuronide isomer 1	1.12 ± 0.03 a	0.72 ± 0.01 c	0.804 ± 0.006 b
11	20.01	350	549	505 (100), 463 (24), 301 (48)	Quercetin-*O*-malonylhexoside	0.580 ± 0.007 b	0.76 ± 0.03 a	0.77 ± 0.02 a
12	21.23	370	549	301 (100)	Quercetin 7-O-malonylhexoside	0.96 ± 0.01	nd	nd
13	23.00	335	445	269 (100)	Apigenin-*O*-glucuronide	0.73 ± 0.01 a	0.67 ± 0.04 c	0.712 ± 0.002 b
14	24.71	343	533	489 (100), 285 (100)	Luteolin-*O*-malonylhexoside	1.21 ± 0.07 a	0.733 ± 0.004 b	nd
15	27.67	340	701	539 (23), 377 (100), 307 (40), 275 (32)	Oleuropein glucoside	1.27 ± 0.04 b	0.511 ± 0.002 a	1.16 ± 0.01 c
16	28.51	282	685	493 (100), 337 (21)	Luteolin-7-*O*-β-*D*-Glucopyranoside	0.473 ± 0.006	nd	nd
17	29.70	335	609	563 (100), 285 (42)	Luteolin-6,8-di-*C*-hexoside	1.34 ± 0.05 a	0.046 ± 0.003 b	1.3 ± 0.1 a
					TPA	1.19 ± 0.02 c	10.5 ± 0.3 a	1.53 ± 0.08 b
					TF	9.27 ± 0.05 a	5.36 ± 0.07 c	6.5 ± 0.2 b
					THT	-	-	1.27 ± 0.01
					TPC	10.46 ± 0.07 b	15.8 ± 0.3 a	9.3 ± 0.1 c

nd: not detected; TPA: total phenolic acids; TF: total flavonoids; THT: total hydrolysable tannins; TPC: total phenolic compounds. Calibration curves used in the quantification were standard calibration curves: chlorogenic acid (y = 168,823x − 161,172, *R*^2^ = 0.999, LOD = 0.20 µg/mL and LOQ = 0.68 µg/mL, peaks 1, 2 and 5); ellagic acid (y = 26,719x − 317,255, *R*^2^ = 0.9986, LOD = 41.20 µg/mL and LOQ = 124.84 µg/mL, peak 3); *p*-coumaric acid (y = 301,950x + 6966.7, *R*^2^ = 0.9999, LOD = 0.68 µg/mL and LOQ = 1.61 µg/mL, peak 4); apigenina-7-*O*-glucósido (y = 10,683x − 45,794, *R*^2^ = 0.996, LOD = 136.95 µg/mL and LOQ = 414.98 µg/mL, peaks 6 and 13–16); quercetin-3-*O*-glucoside (y = 34,843x − 160,173, *R*^2^ = 0.9998, LOD = 0.21 µg/mL and LOQ = 0.71 µg/mL, peaks 7 and 9–12); caffeic acid (y = 388,345x + 406,369, *R*^2^ = 0.999, limit of detection (LOD) = 0.78 µg/mL and limit of quantitation (LOQ) = 1.97 µg/mL, peak 8); and apigenin-6-*C*-glucoside (y = 107,025x + 61,531, *R*^2^ = 0.9989, LOD = 0.19 µg/mL and LOQ = 0.63 µg/mL, peak 17). Means in the same row followed by different Latin letters are significantly different at *p* < 0.05 according to Tukey’s HSD test. Significant differences (*p* < 0.001) between two samples were assessed by Student’s *t*-test.

**Table 8:** Retention time (Rt), wavelengths of maximum absorption in the visible region (λmax), mass spectral data, tentative identification, and quantification (mg/g of extract) of the phenolic compounds present in the hydroethanolic extracts of leaves, decoctions, and hydroethanolic extracts of leaves of *Sonchus oleraceus* after decoction (mean ± SD, *n* = 3).

						Quantification		
Peak	Rt (min)	λmax (nm)	[M–H]^−^ (*m*/*z*)	MS^2^ (*m*/*z*)	Tentative Identification	Hydroethanolic Extracts	Decoctions	Hydroethanolic Extracts after Decoction
1	4.52	328	311	179 (85), 149 (54), 135 (100)	Caftaric acid	0.72 ± 0.02 a	0.060 ± 0.003 b	tr
2	5.95	292sh342	465	447 (5), 375 (10), 357 (8), 345 (100), 257 (15), 241 (42)	Dihydroquercetin 6-*C*-hesoxide	0.68 ± 0.04 a	0.556 ± 0.001 b	0.482 ± 0.001 c
3	6.39	325	353	191 (100), 179 (6), 161 (5), 135 (4)	*cis*-5-*O*-Caffeoylquinic acid	1.45 ± 0.06 c	0.314 ± 0.005 a	0.215 ± 0.005 b
4	6.95	324	353	191 (100), 179 (4), 161 (5), 135 (3)	*trans*-5-*O*-Caffeoylquinic acid	1.21 ± 0.06 c	0.277 ± 0.007 a	0.191 ± 0.006 b
5	8.15	320	431	413 (5), 385 (100), 341 (3), 311 (10)	Apigenin-6-*C*-glucoside	0.55 ± 0.04 a	0.27 ± 0.01 b	0.031 ± 0.001 c
6	12.80	356	631	479 (5), 317 (6), 271 (25)	Myricetin-*O*-(*O*-galloyl)-hexoside	7.36 ± 0.09 a	0.509 ± 0.002 b	nd
7	13.24	270sh342	623	447 (20), 285 (100)	Kaempferol-*O*-glucuronyl-*O*-hexoside	6.6 ± 0.3 a	0.57 ± 0.01 b	0.509 ± 0.003 c
8	15.55	334	609	285 (100)	Kaempferol-*O*-hexoside-*O*-hexoside	0.78 ± 0.02 a	nd	0.489 ± 0.001 b
9	15.77	285	449	287 (20), 269 (100), 225 (2), 209 (2), 151 (27)	Eriodictyol-hexoside isomer 1	tr	tr	tr
10	16.30	285	449	287 (21), 269 (100), 223 (7), 209 (2), 177 (22)	Eriodictyol-hexoside isomer 2	0.78 ± 0.03	tr	tr
11	18.59	347	461	285 (100)	Luteolin-*O*-glucuronide	7.2 ± 0.3 a	2.5 ± 0.1 c	3.1 ± 0.1 b
12	22.80	335	445	269 (100)	Apigenin-*O*-glucuronide	34 ± 2 a	2.8 ± 0.1 c	3.92 ± 0.09 b
13	23.87	334	445	269 (100)	Apigenin-*O*-glucuronide	10.1 ± 0.5	nd	nd
14	27.19	333	445	269 (100)	Apigenin-*O*-glucuronide	2.1 ± 0.1	nd	nd
					TPA	3.376 ± 0.02 a	0.651 ± 0.01 b	0.406 ± 0.01 c
					TF	69.6 ± 0.4 a	6.8 ± 0.2 c	7.8 ± 0.2 b
					TPC	73.0 ± 0.4 a	7.5 ± 0.2 c	8.2 ± 0.2 b

nd: not detected; tr: traces; TPA: total phenolic acids; TF: total flavonoids; TPC: total phenolic compounds. Calibration curves used in the quantification were standard calibration curves: caffeic acid (y = 388,345x + 406,369, *R*^2^ = 0.999, limit of detection (LOD) = 0.78 µg/mL and limit of quantitation (LOQ) = 1.97 µg/mL, peak 1); quercetin-3-*O*-glucoside (y = 34,843x − 160,173, *R*^2^ = 0.9998, LOD = 0.21 µg/mL and LOQ = 0.71 µg/mL, peaks 2 and 6–8); chlorogenic acid (y = 168,823x − 161,172, *R*^2^ = 0.999, LOD = 0.20 µg/mL and LOQ = 0.68 µg/mL, peaks 3 and 4); apigenin-6-*C*-glucoside (y = 107,025x + 61,531, *R*^2^ = 0.9989, LOD = 0.19 µg/mL and LOQ = 0.63 µg/mL, peak 5); naringenin (y = 18,433x + 78,903, *R*^2^ = 0.9998, LOD = 18.66 µg/mL and LOQ = 56.55 µg/mL, peaks 9 and 10); and apigenina-7-*O*-glucósido (y = 10,683x − 45,794, *R*^2^ = 0.996, LOD = 136.95 µg/mL and LOQ = 414.98 µg/mL, peaks 11–14). Means in the same row followed by different Latin letters are significantly different at *p* < 0.05 according to Tukey’s HSD test. Significant differences (*p* < 0.001) between two samples were assessed by Student’s *t*-test.

**Table 9:** Retention time (Rt), wavelengths of maximum absorption in the visible region (λmax), mass spectral data, tentative identification, and quantification (mg/g of extract) of the phenolic compounds present in the hydroethanolic extracts of leaves, decoctions, and hydroethanolic extracts of leaves of *Sonchus asper* after decoction (mean ± SD, *n* = 3).

						Quantification		
Peak	Rt (min)	λmax (nm)	[M–H]^−^ (*m*/*z*)	MS^2^ (*m*/*z*)	Tentative Identification	Hydroethanolic Extracts	Decoctions	Hydroethanolic Extracts after Decoction
1	4.35	328	311	179 (85), 149 (54), 135 (100)	Caftaric acid	1.48 ± 0.02 a	0.580 ± 0.003 b	tr
2	5.98	292sh342	465	447 (5), 375 (10), 357 (8), 345 (100), 257(15), 241 (42)	Dihydroquercetin 6-*C*-hesoxide	0.280 ± 0.005 a	0.175 ± 0.001 b	0.106 ± 0.001 c
3	6.41	325	353	191 (100), 179 (6), 161 (5), 135 (4)	*cis*-5-*O*-Caffeoylquinic acid	2.13 ± 0.02 a	1.16 ± 0.06 c	0.139 ± 0.005 b
4	7.01	324	353	191 (100), 179 (4), 161 (5), 135 (3)	*trans*-5-*O*-Caffeoylquinic acid	3.4 ± 0.1 a	0.79 ± 0.03 b	0.167 ± 0.006 c
5	8.30	320	431	413 (5), 385 (100), 341 (3), 311 (10)	Apigenin-6-*C*-glucoside	0.80 ± 0.05 b	1.00 ± 0.01 a	0.55 ± 0.03 c
6	12.02	328	473	311 (90), 293 (90)	Caffeoyl hexosylpentoside	0.23 ± 0.01 a	0.038 ± 0.002 b	nd
7	12.76	278	451	241 (20), 307 (5), 289 (6)	(Epi)catechin-*O*-glucoside	13.8 ± 0.6 a	0.241 ± 0.007 b	nd
8	13.40	277	451	241 (20), 307 (5), 289 (6)	(Epi)catechin-*O*-glucoside	10.6 ± 0.2 a	1.20 ± 0.03 b	0.178 ± 0.007 c
9	14.29	328	473	311 (100), 293 (90), 219 (5), 179 (10), 149 (3), 135 (3)	*cis*-Chicoric acid	0.038 ± 0.002 b	0.18 ± 0.01 a	0.016 ± 0.001 c
10	15.37	328	473	311 (100), 293 (90), 219 (5), 179 (10), 149 (3), 135 (3)	*trans*-Chicoric acid	0.017 ± 0.001	nd	tr
11	18.59	347	461	285 (100)	Luteolin-*O*-glucuronide	1.35 ± 0.06 a	0.32 ± 0.02 c	0.36 ± 0.01 b
12	22.88	335	445	269 (100)	Apigenin-*O*-glucuronide	34.6 ± 0.7 a	3.7 ± 0.2 c	4.9 ± 0.1 b
13	29.09	348	609	357 (100), 327 (98)	Luteolin-6-*C*-(6-*O*-hexosyl)hexoside	0.217 ± 0.001 a	0.114 ± 0.003 b	0.103 ± 0.002 c
					TPA	7.3 ± 0.1 a	2.75 ± 0.02 b	0.270 ± 0.01 c
					TF	62 ± 1 a	6.7 ± 0.2 b	6.2 ± 0.1 c
					TPC	69 ± 2 a	9.5 ± 0.2 b	6.4 ± 0.1 c

nd: not detected; tr: traces; TPA: total phenolic acids; TF: total flavonoids; TPC: total phenolic compounds. Calibration curves used in the quantification were standard calibration curves: caffeic acid (y = 388,345x + 406,369, *R*^2^ = 0.999, limit of detection (LOD) = 0.78 µg/mL and limit of quantitation (LOQ) = 1.97 µg/mL, peaks 1, 6, 9 and 10); quercetin-3-*O*-glucoside (y = 34,843x − 160,173, *R*^2^ = 0.9998, LOD = 0.21 µg/mL and LOQ = 0.71 µg/mL, peak 2); chlorogenic acid (y = 168,823x—161,172, *R*^2^ = 0.999, LOD = 0.20 µg/mL and LOQ = 0.68 µg/mL, peaks 3 and 4); apigenin-6-*C*-glucoside (y = 107,025x + 61,531, *R*^2^ = 0.9989, LOD = 0.19 µg/mL and LOQ = 0.63 µg/mL, peaks 5, 11 and 13); catechin (y = 84,950x—23,200, *R*^2^ = 1, LOD = 0.44 µg/mL and LOQ = 1.33 µg/mL, peaks 7 and 8); and apigenina-7-*O*-glucósido (y = 10,683x—45,794, *R*^2^ = 0.996, LOD = 136.95 µg/mL and LOQ = 414.98 µg/mL, peak 12). Means in the same row followed by different Latin letters are significantly different at *p* < 0.05 according to Tukey’s HSD test. Significant differences (*p* < 0.001) between two samples were assessed by Student’s *t*-test.

**Table 10:** Antioxidant activity, cytotoxicity, and anti-inflammatory activities of the hydroethanolic extracts of leaves, decoctions, and hydroethanolic extracts of leaves after decoction (mean ± SD, *n* = 3).

Antioxidant Activity		S1 *	S2	S3	S4	S5	S6	Trolox
TBARS (EC_50_; µg/mL) ^a^	Hydroethanolic extracts	147 ± 2 c	147 ± 4 c	142 ± 4 c	131 ± 3 c	120 ± 4 c	144 ± 1 c	
	Decoctions	304 ± 2 a	298 ± 5 b	295 ± 9 b	287 ± 9 b	286 ± 6 b	281 ± 4 b	5.4 ± 0.3
	Hydroethanolic extracts after decoctions	323 ± 5 b	341 ± 1 a	330 ± 6 a	327 ± 3 a	309 ± 3 a	318 ± 6 a	
OxHLIA (IC_50_; µg/mL) ^a^ Δ*t* = 60 min	Hydroethanolic extracts	42 ± 1 c	42 ± 1 b	22 ± 1 b	22 ± 1 b	19 ± 1 c	35 ± 3 c	19.6 ± 0.7
	Decoctions	55 ± 2 b	143 ± 6 a	161 ± 2 a	141 ± 4 a	51 ± 2 b	49 ± 4 b	
	Hydroethanolic extracts after decoctions	89 ± 9 a	na	166 ± 8 a	143 ± 3 a	70 ± 3 a	62 ± 2 a	
Δ*t* = 120 min	Hydroethanolic extracts	69 ± 2 c	63 ± 2 b	63 ± 2 c	63 ± 2 b	52 ± 1 c	112 ± 9 b	41 ± 1
	Decoctions	99 ± 6 b	255 ± 13 a	243 ± 2 b	214 ± 6 a	98 ± 6 b	116 ± 9 b	
	Hydroethanolic extracts after decoctions	253 ± 19 a	na	258 ± 14 a	209 ± 7 a	126 ± 4 a	130 ± 6 a	
Cytotoxicity to tumor cell lines (GI_50_ μg/mL) ^b^								Ellipticine
CaCo2	Hydroethanolic extracts	229 ± 4 b	251 ± 3 b	379 ± 6 b	308 ± 22 b	>400 a	257 ± 1 b	0.20 ± 0.02
	Decoctions	>400 a	>400 a	>400 a	>400 a	>400 a	>400 a	
	Hydroethanolic extracts after decoctions	237 ± 17 b	87 ± 3 c	135 ± 2 c	150 ± 14 c	198 ± 2 b	186 ± 10 c	
NCI-H460	Hydroethanolic extracts	66 ± 7 c	197 ± 16 b	192 ± 2 c	206 ± 17 a	168 ± 18 c	164 ± 17 c	0.249 ± 0.002
	Decoctions	>400 a	>400 a	>400 a	183 ± 2 b	308 ± 27 a	>400 a	
	Hydroethanolic extracts after decoctions	344 ± 11 b	210 ± 13 b	205 ± 4 b	132 ± 10 c	236 ± 12 b	257 ± 23 b	
MCF-7	Hydroethanolic extracts	267 ± 26 b	249 ± 24 b	259 ± 24 b	246 ± 3 b	>400 a	268 ± 12 b	0.251 ± 0.001
	Decoctions	>400 a	>400 a	>400 a	>400 a	362 ± 43 b	>400 a	
	Hydroethanolic extracts after decoctions	226 ± 17 c	226 ± 2 b	223 ± 2 c	237 ± 5 c	246 ± 15 c	235 ± 4 c	
Cytotoxicity to non-tumor cell lines (GI_50_ µg/mL) ^b^								Ellipticine
PLP2	Hydroethanolic extracts	155 ± 13 b	182 ± 7 c	232 ± 13 c	231 ± 12 b	>400 a	231 ± 6 a	6.3 ± 0.4
	Decoctions	>400 a	>400 a	>400 a	260 ± 13 a	206 ± 15 b	178 ± 15 b	
	Hydroethanolic extracts after decoctions	62 ± 2 c	230 ± 5 b	270 ± 16 b	178 ± 19 c	221 ± 6 b	227 ± 6 a	
Anti-inflammatory activity (EC_50_ μg/mL) ^c^								Dexamethasone
RAW 264.7	Hydroethanolic extracts	21 ± 1 c	195 ± 7 a	232 ± 3 c	84 ± 6 a	21 ± 2 c	187 ± 17 a	16 ± 1
	Decoctions	42 ± 2 b	130 ± 4 b	90 ± 9 b	64 ± 2 b	110 ± 5 a	48 ± 3 b	
	Hydroethanolic extracts after decoctions	93 ± 5 a	33 ± 2 c	79 ± 4 c	90 ± 4 a	78 ± 5 b	31 ± 1 b	

* *Cichorium spinosum* L. (S1); *Centaurea raphanina* subsp. *mixta* (DC.) Runemark (S2); *Picris echioides* (L.) Holub (S3); *Urospermum picroides* (L.) Scop. ex. F.W. Schmidt (S4); *Sonchus oleraceus* L. (S5); and *S. asper* L. (S6). na: no activity; ^a^ EC_50_: extract concentration corresponding to 50% of antioxidant activity (TBARS) or IC_50_ values (extract concentration required to keep 50% of the erythrocyte population intact for 60 and 120 min (OxHLIA assay)); ^b^ GI_50_: extract concentration responsible for 50% inhibition of growth of human tumor (AGS, CaCo2, NCI-H400, and MCF-7) or non-tumor cell lines (PLP2); ^c^ EC_50:_ extract concentration responsible for achieving 50% of the inhibition of NO production. Means in the same column and for the same sample followed by different Latin letters are significantly different at *p* < 0.05 according to Tukey’s HSD test. Significant differences (*p* < 0.001) between two samples were assessed by Student’s *t*-test.

**Table 11:** Antibacterial and antifungal activity (mg/mL) of the hydroethanolic extracts of leaves, decoctions, and hydroethanolic extracts of leaves after decoction.

Antibacterial Activity		S1 *	S2	S3	S4	S5	S6	E211	E224
		MIC/MBC	MIC/MBC	MIC/MBC	MIC/MBC	MIC/MBC	MIC/MBC	MIC/MBC	MIC/MBC
*S. aureus*	Hydroethanolic extracts	0.50/1.00	1.00/2.00	0.25/0.50	0.50/1.00	2.00/2.00	0.50/1.00	4.00/4.00	1.00/1.00
	Decoctions	2.00/2.00	2.00/2.00	2.00/2.00	2.00/2.00	2.00/2.00	2.00/2.00		
	Hydroethanolic extracts after decoctions	0.50/1.00	0.25/0.50	0.50/1.00	0.50/1.00	0.50/1.00	0.50/1.00		
*B. cereus*	Hydroethanolic extracts	1.00/2.00	0.50/1.00	0.50/1.00	1.00/2.00	0.50/1.00	1.00/2.00	0.50/0.50	2.00/4.00
	Decoctions	0.50/2.00	0.50/2.00	0.50/2.00	0.50/2.00	0.50/2.00	0.50/2.00		
	Hydroethanolic extracts after decoctions	1.00/2.00	1.00/2.00	0.50/1.00	0.50/1.00	0.50/1.00	0.50/1.00		
*L. monicytogenes*	Hydroethanolic extracts	1.00/2.00	1.00/2.00	1.00/2.00	1.00/2.00	1.00/2.00	1.00/2.00	1.00/2.00	0.50/1.00
	Decoctions	1.00/2.00	1.00/2.00	1.00/2.00	1.00/2.00	1.00/2.00	1.00/2.00		
	Hydroethanolic extracts after decoctions	1.00/2.00	1.00/2.00	1.00/2.00	1.00/2.00	1.00/2.00	1.00/2.00		
*E. coli*	Hydroethanolic extracts	1.00/2.00	1.00/2.00	1.00/2.00	1.00/2.00	1.00/2.00	1.00/2.00	1.00/2.00	0.50/1.00
	Decoctions	1.00/2.00	1.00/2.00	1.00/2.00	1.00/2.00	1.00/2.00	1.00/2.00		
	Hydroethanolic extracts after decoctions	1.00/2.00	1.00/2.00	1.00/2.00	1.00/2.00	1.00/2.00	1.00/2.00		
*S.* *typhimurium*	Hydroethanolic extracts	1.00/2.00	1.00/2.00	1.00/2.00	1.00/2.00	1.00/2.00	1.00/2.00	1.00/2.00	1.00/1.00
	Decoctions	1.00/2.00	1.00/2.00	1.00/2.00	1.00/2.00	1.00/2.00	1.00/2.00		
	Hydroethanolic extracts after decoctions	1.00/2.00	1.00/2.00	1.00/2.00	1.00/2.00	1.00/2.00	1.00/2.00		
*En. cloacae*	Hydroethanolic extracts	1.00/2.00	1.00/2.00	1.00/2.00	1.00/2.00	1.00/2.00	1.00/2.00	2.00/4.00	0.50/0.50
	Decoctions	1.00/2.00	1.00/2.00	1.00/2.00	1.00/2.00	1.00/2.00	1.00/2.00		
	Hydroethanolic extracts after decoctions	1.00/2.00	1.00/2.00	1.00/2.00	1.00/2.00	1.00/2.00	1.00/2.00		
**Antifungal activity**								E211	E224
		MIC/MFC	MIC/MFC	MIC/MFC	MIC/MFC	MIC/MFC	MIC/MFC	MIC/MFC	MIC/MFC
*A. ochraceus*	Hydroethanolic extracts	0.50/1.00	0.50/1.00	0.50/1.00	0.50/1.00	0.50/1.00	0.50/1.00	1.00/2.00	1.00/1.00
	Decoctions	0.50/1.00	0.50/1.00	0.50/1.00	0.50/1.00	0.50/1.00	0.50/1.00		
	Hydroethanolic extracts after decoctions	0.50/1.00	0.50/1.00	0.50/1.00	0.50/1.00	0.50/1.00	0.50/1.00		
*A. niger*	Hydroethanolic extracts	2.00/2.00	1.00/2.00	1.00/2.00	1.00/2.00	1.00/2.00	1.00/2.00	1.00/2.00	1.00/1.00
	Decoctions	1.00/2.00	1.00/2.00	1.00/2.00	1.00/2.00	1.00/2.00	2.00/2.00		
	Hydroethanolic extracts after decoctions	1.00/1.00	1.00/1.00	1.00/1.00	1.00/1.00	1.00/1.00	1.00/1.00		
*A. versicolor*	Hydroethanolic extracts	0.50/1.00	0.25/0.50	0.25/0.50	0.25/0.50	0.25/0.50	0.25/0.50	2.00/2.00	1.00/1.00
	Decoctions	0.50/1.00	0.50/1.00	0.50/1.00	0.25/0.50	0.50/1.00	0.50/1.00		
	Hydroethanolic extracts after decoctions	0.50/1.00	1.00/2.00	1.00/2.00	1.00/2.00	1.00/2.00	1.00/2.00		
*P. funiculosum*	Hydroethanolic extracts	0.25/1.00	0.25/1.00	0.25/1.00	0.25/1.00	0.25/1.00	0.25/1.00	1.00/2.00	0.50/0.50
	Decoctions	0.25/1.00	0.25/1.00	0.25/1.00	0.25/1.00	0.25/1.00	0.25/1.00		
	Hydroethanolic extracts after decoctions	0.25/1.00	0.25/1.00	0.25/1.00	0.25/1.00	0.25/1.00	0.25/1.00		
*P. aurantiogriseum*	Hydroethanolic extracts	1.00/2.00	1.00/2.00	0.50/1.00	0.50/1.00	0.50/1.00	0.50/1.00	2.00/4.00	1.00/1.00
	Decoctions	1.00/1.00	1.00/1.00	1.00/1.00	1.00/1.00	1.00/1.00	1.00/1.00		
	Hydroethanolic extracts after decoctions	0.50/1.00	1.00/1.00	1.00/2.00	0.50/1.00	0.50/1.00	0.50/1.00		
*T. viride*	Hydroethanolic extracts	0.50/1.00	0.50/1.00	0.50/0.50	0.50/0.50	0.50/0.50	0.50/0.50	1.00/2.00	0.50/0.50
	Decoctions	0.50/0.50	0.50/0.50	0.50/0.50	0.50/0.50	0.50/0.50	0.50/1.00		
	Hydroethanolic extracts after decoctions	0.25/0.50	0.25/0.50	0.25/0.50	0.25/0.50	0.25/0.50	0.25/0.50		

* *Cichorium spinosum* L. (S1); *Centaurea raphanina* subsp. *mixta* (DC.) Runemark (S2); *Picris echioides* (L.) Holub (S3); *Urospermum picroides* (L.) Scop. ex. F.W. Schmidt (S4); *Sonchus oleraceus* L. (S5); and *S. asper* L. (S6). MIC—minimum inhibitory concentration; MBC—minimum bactericidal concentration.Regarding the antifungal effects, all the extracts from all the species were effective against the studied fungi, showing lower MIC and/or MFC values than both positive controls, as well as lower MFC values than E211. Moreover, the hydroethanolic extracts before and after decoction of the leaves for all the species were similarly or more effective against *A. versicolor* and *T. viride*, respectively, than the positive controls, while no differences were recorded among the extracts of all the species against *A. ochraceus* and *P. funiculosum*.

## Data Availability

The original contributions presented in the study are included in the article, further inquiries can be directed to the corresponding author.
